# Use of pre-operative blood products in abdominal trauma: a planned secondary analysis of the GOAL-trauma study

**DOI:** 10.1016/j.eclinm.2026.103814

**Published:** 2026-03-04

**Authors:** Wee-Han Ng, Michael F. Bath, Joachim Amoako, Katharina Kohler, Daniel U. Baderhabusha, Eder Cáceres, Carlos M. Nuño-Guzmán, Ramani Moonesinghe, Laura Hobbs, Brandon G. Smith, Thomas G. Weiser, Timothy C. Hardcastle, Zane B. Perkins, Tom Bashford, GOAL-Trauma Collaborative, M.F. Bath, T. Edmiston, B.G. Smith, D. Clarke, A. Kwizera, L. Hobbs, K. Kohler, A. Mazzoleni, F.F.I. Fareed, Z. Zhang, R. Thavayogan, J. Erhabor, O. Mantle, C. Hammer, Z. Perkins, M. Marsden, R. Davenport, R.J. Davies, J. Amoako, R. Moonesinghe, T.G. Weiser, A. Leather, T.C. Hardcastle, R. Naidoo, Y.R.A. Nordín Servín, A. Conway Morris, K. Lakhoo, G.A. Bass, J.M. Wohlgemut, P. Hutchinson, T. Bashford, W.H. Ng, M.F. Bath, K. Kohler, Z.B. Perkins, A. Dogjani, A. Tidjane, S.E. Vélez, C. O'Flynn, F. Haider, A. Litvin, R. Filho, T. Tientcheu, E.G. Wong, R. Wang, J. Wang, Y. Ni, Z. Wang, Z. Tian, M. Fang, M. Zhou, S. Liu, K. Xie, Z. Zhang, X. Guo, Y. Ke, H. Ni, Q. Luo, E. Caceres, L.F. Reyes, L. Pino, D.U. Baderhabusha, B. Cissa Wa Numbe, A. Meselhi, M.M. Elsayed, S. Abdelmohsen, A. Elkhouly, A. Elmorshdy, G. Abouelnagah, A. Osman, H. Taher, W. Shenkutie, M. Senbu, N. Bayleyegn, M. Merene, M. Ahmed, S. Gudugbe, J. Amoako, M. Morna, E. Gyabaah, H. Seidu-Aroza, I. Baloyiannis, K. Bouchagier, F. Mulita, A. Ioannidis, M. Rivera, F. Régis, L. Bains, M. Khajanchi, L. Sartarelli, R. Bollino, M. Fedi, A. Bottari, F. Cammelli, G. Calini, A. Piccolo, D. Visconti, M. Altomare, L. Carenzo, F. Fleres, Y. Iwao, R. Parker, C.K. Tiong, C.X. Teoh, A.D. Zakaria, C.M. Nuño-Guzmán, A. González-Ojeda, C. Wakeman, E. Ikwutah, M. Daniyan, A. Adamu, E. Akpo, I. Chukwu, M. Bashiru, B. Akanni, J. Olaogun, B. Nomayo-Oriabure, H. Abiyere, E. Oriabure, F.F. Khidri, S.A. Naqi, T. Khan, K. Faheem, R.S. Pederzoli, H. Abu-Arish, M. Youssef, C. Huaroto-Landeo, I. Carolino Gomes, N. Gatta, I. Negoi, S. Katorkin, N. Alsubaie, J.T.T. Goo, S. Balasubramaniam, M.S. Hassan, S.A. Mohamed, A.E. Abdishakur, T.C. Hardcastle, R. Naidoo, R. Crawford, M. Moeng, H.J. Kruger, M. Serrano-Navidad, A. Landaluce-Olavarria, C.C. Lopes Moreira, H. Llaquet-Bayo, K. Jayasuriya, J.A.S.B. Jayasundara, D. Subasinghe, J. Mithushan, A. Ibrahim, M. Elnour, I. Ahmed, I. Adel, L. Mohammed, S. Bakhit, M. Elbashier, R. Musa, J. Amin, M. Yassin, A. Babiker, A. Noureldin, A. Abdalazeez, S. Benediktsdottir, L. Hasan, S. Hamad, N. Mansour, O. Homchan, A. Hasnaoui, A. Bouzid, W. Riahi, M. Ergenç, B. Yigit, B. Citgez, M. Yilmaz, Y.F. Aydoğdu, A. Guner, H. Karakullukcu, K. Tuncer, A.N. Sanli, M.T. Demirpolat, Ç. Büyükkasap, A.C. Yildirim, F. Feratoglu, S. Smoliar, H. Roocroft, G. McKnight, M. Hughes, J.V. Taylor, E. Yung, E.J. Nevins, S. Owen-Smith, A. Mian, M. Alfa-Wali, C. Menichetti, T. Jodlowski, S. Mundell, O. Khalil, S. Jay, M. El-Boghdady, P. Pratheepan, A. Abouelnaga, A. Brooks, S. Yoong, Y. Al Azzawi, Y.S. Lim, S. Agarwal, P. Petrone, D. Stephens, N. Starr, A. Teichman, C. Dodgion, C. Wolff, T. Egodage, J. Brady, J. Brown, D. Leon, A. Pienovi, R. Saleh, K. Doçi, E. Bregaj, J. Mansouri, B. Tabeti, M. Titarelli, M.M. Avalos Barraza, M. Sánchez, E. Caldani, A. Giavarini, C. Groombridge, E. Ban, A. Abdulla, V. Bereshchenko, P. Tereshchenko, I. Marcos, R. Lima, N. Nwenasi, E. Aloys, H. Uchino, J.R. Grushka, W. Davalan, C. Chen, F. Ge, K. Lu, J. Zhang, X. Liu, X. Li, Z. Qi, N. Wang, J. Tang, S. Wang, F. Gao, Y. Lu, H. Du, C. Wu, H. Fu, J. Liu, T. Chen, M. Du, J. Guan, Q. Lu, Y. Li, J. Li, Q. Wang, W. Luo, K. Wang, H. Zhang, J. Dong, S. Gelvez, K. Reyes, D. Tsongo, J. Muhoza, A. Mirindi, J.R. Birindwa, H. Aboelfadl, A. Elasad, S. Elsheikh, F. Elsaied, M. Atef, R. Elnour, N. Elbaloula, E.A. Shanab, M. Adres, O. Mohamed, S. Adam, O. Younes, T. Elboraay, O. Abdelfattah, M.A. Elfadali, A. Jader, A. Ziada, K. Sarhan, M. Sherif, R. Gomaa, R. Mohamed, E. Fouda, A. Elshaboury, M. Alshraiedeh, H. Elhadidi, A. Eldiasti, M. Ahmed, E. Elkoury, H. Abdelhady, M. Amasha, A. Elbadrawy, M. Nassif, M. Hamed, D. Zahran, S. Abdelaal, O.S. Abdelfattah, W. Shehada, M. Alsharif, M. Elnadi, O. Sharaf, M. Elgliand, M. Badr, A. Hegazi, A. Gohar, A. Elshal, M. Abdelhady, M. Saadawi, M. Mohammed, E.Y. Salem, M. Madany, H. Mansour, A. Ashour, A. AbuSuliman, K. Tolba, M.S. Elgendy, M. Ezz, M. Marei, H.I. Taha, I. Younes, A. Abouammar, R. Wael, Asmaa Elmorshdy, Aesha Elmorshdy, A.M. Ibrahim, A. El-Borollosy, S. Elmorshdy, A. Mohamed, A. Adel, N.H. El-Saeed, M. Essam, E.A.K. Abdelraheem, M. Shaapan, M. Salah, E. Saber, M.A. Ibrahim, R. Mamdouh, A.M.M. Mohamadin, F. Moharb, O. Shaqran, Z. Selim, Y. Tanas, M. Khalil, B. Eldin, Y. Gaber, A. Ibrahim, D. Bekhit, B. Mohamed, A. Farrag, M. Saadawi, H. Mahfouz, N. Sayed, M. El Mahrouki, A. Amgad, D. Elmagdoub, S. Paulo, B. Gebremedhin, A. Eticha, B. Bayissa, K. Urgessa, B. Tasew, Y. Yilma, L.G. Mude, O. Tilahun, L. Buta, A.G. Mideksa, T. Gemechu, A. Tilahun, Y. Degefu, F. Quenin, E. Acquah, I. Abdull-Karim, C. Takyi, G. Aryee, T. Wordui, A. Bowan, P. Kumassah, N. Adu-Aryee, N. Naalane, F. Dedey, J. Nsaful, O. Ekor, G. Rahman, M. Nortey, R. Baidoo, M. Amoako-Boateng, D. Enti, K. Agyen-Mensah, E. Quartson, T. Agyen, E. Ofori, P. Mensah, V. Kudoh, D. Arthur, P. Maison, F. Akum, F. Owusu, N. Affram, D. Tamatey, C. Sarakatsianou, D. Papaspyrou, A. Antzoulas, K. Kitsou, V. Garantzioti, V. Leivaditis, A. Vouchara, K. Katsiafliaka, S. Morales, E. Galindo, A. Meza, M. Colón, E. Cardona, K. Louis, R. Osias, C. Lominy, A. Capois, S.A. Khan, V. Verma, S. Amin, A. Gaikwad, V. Tonini, M. Cervellera, M. Fumagalli, M. Zizzo, D. Luppi, H. Yu, L. Di Donato, F. Leo, C. Cecchi, G. Ripamonti, B. Pesi, L. Piombetti, M. Pagani, G. Pascale, S. Di Salvatore, C. Tasca, S. Giannessi, R. De Vincenti, E. Monati, F. Renzi, L. Vacca, F. Matarazzo, D. Perini, A. Di Bella, L. Fortuna, M. Rottoli, M. Binetti, M. Tescione, G. Sera, N. Pellicano, S. Pangallo, E. Ballauri, M. Santarelli, S. Cimbanassi, S. Cioffi, G. Curreri, M. Ceolin, D. Del Fabbrio, S. Giudici, M. Cecconi, T. Sinicropi, C. Mazzeo, K. Sato, K. Otoki, D. Baraka, R.M.Z. Ang, M.N.F. Zulkifli, K.C. Sheng, M.T.Y. Wong, N. Aziz, P.J.H. Lim, C.C.E. Koay, Y.X. Teoh, I. Chik, Z. Zakaria, M.H.S. Satar, M.R. Mazlan, L. Bravo-Cuéllar, J. Orozco-Camacho, A. Nava-Franco, M. Ibarra-Tapia, F. López-Ortega, F. Romo-Pérez, R. Contreras-Arias, M. Alejo-Rivera, S.J. Vázquez-Sánchez, C. Fuentes-Orozco, A. McCombie, J. Dasril, Y. Teo, A.A. Fagbenro, K. Shafer, L. Iji, S. Gana, M. Bashir, A. Ajayi, I. Gundu, G.D. Mukoro, L. Ukwubile, C. Okeke, A. Jimoh, V. Nduka, K. Bwala, A. Ningi, S. Oriakhi, H. Odion-Obomhense, S. Ekpemo, K. Okpokiri, A.A. Makama, H. Aminu, Z. Ahmad, M. Mustapha, O. Segunfunmi, E. Boladuro, U. Eni, C. Obi, N.L. Kwentoh, D. Idowu, M. Magbagbeola, O. Adolphus, E. Oriabure, S.O. Fatudimu, O. Oloruntoba, R. Eghonghon, S. Omorogbe, S. Shaikh, A.K. Narsani, I. Ujjan, A. Munir, A.I. Memon, F. Hameed, S. Khatoon, A. Talpur, S. Kumar, A. Yousfani, N. Dal, S. Naz, M. Akbar, A.M. Bhatti, N. Amir, S.A. Khaskheli, N. Iqbal, A. Aamir, G. Shamsi, G. Awais, I. Tasleem, N. Iodhi, I. Ahmed, R. Fatima, S. Asif, H. Haroon, A. Jawaid, J. Muneer, H. Ahmed, M. Washdil, A. Hilal, M. Ishaq, S. Ialani, Y. Kumar, M.N. Shehzad, S. Nadeem, N. Ahmed, S. Ahmed, S. Gulzar, G. Khan, Z. Jaffer, A. Gul, M. Khan, A. Faraz, M. Obaid, H. Pirhay, K. Faheem, M. Shafique, R. Nafees, L.C. Soares Barboza de Toledo, A.L. Silva de Sousa, B. Qneiby, R. Jabari, R. Farash, M. Oweidat, R. Matar, M. Shaldan, C. Picasso-Arias, C. Strong, F. Feliciano, L dos Santos, D. Monteiro, S. Alves, D. da Cruz, B. Oprita, E. Dumitru, L. Lichman, O. Davydova, P. Andreev, R. Alyahya, A.M. Alrwais, N.H. Almadi, N. AlShahwan, M. Aladawi, S.H. Aldeligan, A. Alotaibi, J. Lee, S. Gunasekaran, M.W. Ong, D.J.K. Lee, W.W. Lim, L.T. Teo, R.L. Tan, A.S. Hashi, A.A. Omar, A.N. Mohamed, A.M. Abdi, A.H. Salad, A.E. Abdishakur, F. Ganchi, S. Naidoo, K. Moodley, H. Wain, N. Reddy, N. Laher, D. Wineberg, R. Pretorius, R. Pswarayi, E. Laney, O. Lusawana, A. Mushtaq, I. Bogiages, F. Viljoen, F. Mohammed, G. Jacks, L. Mohlala, C. Nyatsambo, S. Mathibela, A. Nortje, S. Makhadi, T. Pratt, K. de Kock, M.Q. Patel, M. Parker, J.J.P. Buitendag, G.V. Oosthuizen, C. Martínez de Carneros, S. Quinto Llopis, L. Cruzado, A. Sainz-Lete, B. Estraviz-Mateos, J.C. Zevallos-Quiroz, I. Augusto Ponce, A. Garcia Domínguez, A. Lizarazu Perez, A. Rodriguez Gonzalez, A. Muñoz-Campaña, A. Campos-Serra, L. Bandara, K. Gunasekara, G. Jayarathne, Y. Arachchi, M. Priyangani, S. Wimalge, R.S.C. Desman, K. Gunarathne, G. Wimalasena, V. Rohana, S. Ranathunga, S. Harikrishanth, J. Jeyaruban, M. Mohammed, A. Mohamed, L. Zeinalabedeen, A. Mahmoud, M. Mohamed, M. Eltahir, G. Ahmed, M. Ahmed, I.A.O. Mohammed, S.A.A. Ibrahim, E.A.H. Aziz, M. Homida, F.S. Mahdi, M. Issak, A. Mohammed, M. Hafiz, H. Makki, N. Awad, A. Elhassan, M. Amin, A. Daffalla, A. Omer, M. Alhadi, M. Mostafa, O. Eljizoly, A. Musa, M. Abdallah, G. Fakhri, A. Ahmed, A. Mohammed, M. Kollind, S. Marchesi, A. Aldirani, A. Almahjaa, M. Abdulkareem, E. Kallas, A. Alfandi, A. Hejazi, B. Alnaser, A. Alnaser, J. Sandouk, S. Sara, K. Ballan, H. Joha, S. Kassis, W. Abboud, M. Ahmad, A. Hamdan, K. Chandacham, T. Jirapongcharoenlap, N. Chotirosniramit, R. Trigui, O. Gaidi, A. Saidani, A. Belhaj, H. Zebda, A. Menif, R. Khelili, Ç. Bayır, Ö. Acar, E. Bozlakoğlu, E. Yavuz, G. Alici, S. Meric, N. Bugdayci, A. Sayar, A. Ergin, A. Saylar, A. Barcin, Y. Altinel, O. Gulcicek, O. Cakir, H. Ozsahin, C. Ersavas, G.K. Aydoğdu, K. Saraçoğlu, N. Dundar, K. Eyuboglu, R. Tekcan, M. Bodur, M. Aktas, B. Erdem, A. Calik, A. Kodalak, A. Oruc, M. Usta, B. Alkas, M. Rahimi, A. Cekic, D. Pehlivan, I. Rizaoglu, A. Mwinyi, B. Canakci, M. Shehada, S. Topaloglu, A. Karaaslan, G. Ercan, Y. Poyrazoğlu, M. Çuhadar, Ö. Özkan, R. Ağcabay, G. Tuncer, S. Farsak, C. Tuğmen, N. Polat, E. Kebapçı, M. Yıldırım, N. Göret, M. Gündal, S. Ünlü, E. Tekel, A. Özpek, H. Tosun, B. Yeşilova, K. Dikmen, H. Göbüt, A. Yavuz, S. Zeren, Y. Sönmez, T. Gulsen, M. Zenciroglu, P. Kyrylo, P. Kostiantyn, P. Ivan, P. Orchard, J. Fyfe, H. Dowell, O. Braun, M. Creed, P. Strong, F. Sweeney, N. Mitchell, I. McClure, D. Parry, O. Gbadegesin, E. Carrington-Windo, M. McKenna, S. Mundell, L. Hall, S. Gasson, E. Crudge, A. Eglinton, R. Davenport, P. Vulliamy, Z.B. Perkins, O. Ugas, L. Holt, H. Jenkinson, J. Tan, G. Ramsay, O. Adepoju, R. Cummine, S. Tariq, A. Mohammad, L. Wilson, A. Musbahi, R. Coates, S.J. Horne, N. Preda, F. Luvisetto, Z. Zhang, I. Saqib, G. Matzakanis, P. Pearce, N. Sánchez-Thompson, C. Scurr, A. Bernstein, M.S. Gonsalves, M.S. Hoque-Uddin, I. Abbott, O. Dada, S. Jamil, H. Read, D. Horner, R. Doonan, A. Stafford, C. Battle, R. Thavayogan, O. Quinn, M. Powar, S. Ling, S. Gourgiotis, H. Shinwari, S. Mohandas, M. Eldoadoa, R. Ismail, G. Melia, N. Gandhi, L. Blackburn, T. Merchant, J. Robinson, S. Mackie, Y.S. Wong, Q. Lee, D. Moris, C.P. Nicholson, S. Provencher, J. Cook, G. Baltazar, K. Cordero-Bermudez, L.E. Walker, M.K. Abou Chaar, R. Koch, K. Faktor, A. Chang, Z. Englert, C. Kyaw, N. Pirozzi, L. Moko, B. Chernock, E. Marshall, J.A. Gellings, J. Krizo, J. Molinari, E. Hancin, I. Armento, P. Hu, R. Uhlich, E. Barnes, A. Rawal, O. Falade, D. Nishijima, D. Leshikar, E. Delgado, S. Al Wageeh, A. Al Yafrosi, L. Minthor, S. Cardelli, W. Chiew Meng, S. Johan, I.E. Ihedoro, A.M. Waryah, D. Chavez, T. Manivannan, M.A.M. Abdalla, A. Itaimi, T.K. Uprak, M. Ulusahin, C.V. Riley, H. Hussein, L. Nicol

**Affiliations:** a International Health Systems Group, Department of Engineering, University of Cambridge, Cambridge, UK; b University of Ghana Medical School, Accra, Ghana; c Department of Surgery, Korle Bu Teaching Hospital, Accra, Ghana; d Department of Anaesthesia, Cambridge University Hospitals NHS Foundation Trust, UK; e Hôpital de Kyeshero, Goma, North Kivu, Democratic Republic of the Congo; f Critical Care Department, Clínica Universidad de la Sabana, Chía, Cundinamarca, Colombia; g Hospital Civil de Guadalajara Fray Antonio Alcalde, Guadalajara, Jalisco, México; h Centro Universitario de Ciencias de la Salud, Universidad de Guadalajara, Guadalajara, Jalisco, México; i Centre for Peri-Operative Medicine, Research Department for Targeted Intervention, University College London, London, UK; j Department of Anaesthesia, East & North Hertfordshire NHS Trust, UK; k NIHR Global Health Research Group on Acquired Brain and Spine Injury, Cambridge, UK; l Department of Surgery, Stanford University, Palo Alto, CA, USA; m Trauma and Burns Unit, Inkosi Albert Luthuli Central Hospital, KwaZulu-Natal Department of Health, Durban, 4058, South Africa; n Department of Surgical Sciences, Nelson R Mandela School of Clinical Medicine (NRMSM), University of KwaZulu-Natal, Durban, 4000, South Africa; o Centre for Trauma Sciences, Blizard Institute, Queen Mary University of London, UK; p Major Trauma Service, Royal London Hospital, Barts Health NHS Trust, London, UK

**Keywords:** Trauma, Blood products, Global surgery

## Abstract

**Background:**

Haemorrhage remains the leading cause of death in patients with abdominal trauma. However, variation remains globally in the standards of transfusion practice. The aim of this study was to characterise global pre-operative transfusion practices for patients undergoing trauma laparotomy and identify any associations between transfusion strategies and mortality.

**Methods:**

This was a planned secondary analysis of the Global Outcomes After Laparotomy for Trauma (GOAL-Trauma) study, an international multicentre prospective observational study conducted at 187 hospital centres across 51 countries. Patients of any age with a blunt or penetrating traumatic injury who underwent a laparotomy within 5 days of presentation were eligible. Patients were excluded if they were undergoing a repeat laparotomy at the recruiting centre within 30 days of the index procedure. Eligible participants were recruited between April 1, 2024, and Dec 31, 2024 during select 30-day periods and were followed up until discharge, death, or 30 days post-operatively (if still hospitalised), whichever came first. Pre-operative blood product usage was recorded from time of injury until index procedure. Countries were stratified by Human Development Index (HDI) and the primary outcome was post-operative in-hospital 30-day mortality. Comparative regression analyses between blood component groups were performed, comparing high versus low transfusion ratios of fresh frozen plasma (FFP) to packed red blood cells (PRBC). The GOAL-Trauma study was registered with ClinicalTrials.gov, NCT06180668.

**Findings:**

Overall, 721 (40.8%) of 1768 patients received any type of pre-operative blood product. Those in the upper HDI tertile received the highest proportions of blood components, across PRBC, FFP, and platelets (p < 0.0001). Whole blood usage was nearly double in the lower HDI tertile compared to middle and upper HDI tertiles (p < 0.0001). Tranexamic acid use was low across all HDI tertiles (529 of 1768 patients, 29.9%). No difference in overall 30-day post-operative mortality risk was observed between those in the high-ratio and low-ratio FFP:PRBC cohorts (OR = 1.52, CI: 0.89–2.64).

**Interpretation:**

Our findings show significant disparity in the usage of pre-operative blood products for trauma patients globally. With ongoing equipoise regarding the optimum balance of blood products for pre-operative resuscitation in trauma, this work informs future research to support the development of global guidelines for blood transfusion practices in trauma and highlights the need for reciprocal learning across income settings.

**Funding:**

Royal College of Surgeons Ratanji Dalal Research Fellowship and the Engineering and Physical Sciences Research Council.

**Research in context**

**Evidence before this study**

We searched OVID Medline and Embase databases for papers published between Jan 1, 2010, and April 7, 2025, on trauma resuscitation strategies and mortality. We found that the majority of studies detailing transfusion strategies and practices in trauma derived from high-income countries. There remains little to no evidence on current transfusion practices in low-income and middle-income countries, where logistical and financial barriers often limit the type and amount of blood products received. Notably, there has been no multicentre international study reporting blood transfusion strategies in patients with traumatic injuries. We aimed to address this knowledge gap.

**Added value of this study**

This planned secondary analysis of the Global Outcomes After Laparotomy for Trauma (GOAL-Trauma) study demonstrates significant disparities in blood product use, with higher use of component therapy in the higher resource settings and a greater use of whole blood in the lower resource settings. Whilst relatively low in cost, the use of tranexamic acid remained low across all settings. Of interest, our findings were suggestive of a high ratio of FFP to PRBC, which included whole blood, being associated with lower mortality at higher transfusion volumes, however careful interpretation is required due to casemix and inherent biases.

**Implications of all the available evidence**

This study demonstrates substantial variation in transfusion strategies worldwide in patients undergoing trauma laparotomy. With whole blood being increasingly adopted globally as the potential best standard of care, a broader global review on transfusion strategies will allow for bidirectional learning for all countries, leading to the development of context-appropriate blood transfusion protocols that can benefit trauma patients globally.

## Introduction

Trauma is a leading cause of mortality globally, with around 4.4 million deaths occurring worldwide every year due to injury^1^ and high rates of associated morbidity and disability. However, it is estimated that around 2 million lives could be saved and 52 million disability-adjusted life years could be averted if current standards of trauma care in low- and middle-income countries (LMICs) were matched to those in high-income countries (HICs).^2^^,^^3^ Trauma care encompasses every part of patient management from time of injury through to rehabilitation, therefore identifying and improving unwarranted variation in every aspect of care is essential if global trauma care standards are to be raised.^4^^,^^5^

Across all trauma victims, around 10% will sustain an abdominal injury.^6^, ^7^, ^8^ Haemorrhage remains the leading cause of death in patients with abdominal trauma,^9^ and for those with evidence of uncontrolled haemorrhage, a trauma laparotomy is a life-saving intervention.^10^ As such, ensuring optimal pre-operative resuscitation is essential. Indeed, attempts to improve patient outcomes in trauma in recent years have focused on optimising fluid resuscitation practices, however significant variation remains globally in the current standards of practice^11^ and a global consensus is still lacking.^12^ In addition, the use of tranexamic acid has been shown to improve mortality rates in trauma patients,^13^ and as an inexpensive heat-stable medicine with a long shelf-life, uptake globally should be high. However, despite being recommended as an essential medicine by the World Health Organisation,^14^ its current use worldwide following traumatic injury remains unclear.

Previous studies have suggested that component therapy using a 1:1:1 or 1:1:2 balanced blood product ratio (fresh frozen plasma (FFP) to platelets to packed red blood cells (PRBC)) improves 6-h survival rates in trauma patients.^15^^,^^16^ These studies have been predominantly conducted in high-resource settings, however component therapy is not achievable in many lower-resource settings due to a combination of cultural, resource, and financial constraints.^17^ The use of whole blood (WB) for transfusion, which is more readily available in lower resource settings predominantly due to practicalities of storage, has been gaining traction within the medical literature in recent years as potentially superior to component therapy.^18^, ^19^, ^20^ This is supported by long-standing WB use in both military trauma care and humanitarian emergencies, where logistical constraints can mirror those in resource-limited settings.^21^ However, there remains equipoise as to the optimum transfusion strategy in trauma and there is a clear need for resource-stratified guidelines that support best practice on a global scale.

The Global Outcomes After Laparotomy for Trauma (GOAL-Trauma) study was an international multicentre prospective observational cohort study of patients undergoing trauma laparotomy.^22^ The study demonstrated that substantial global variation exists across all phases of trauma care, and that despite less severe injury, patients in lower resource settings experienced a higher mortality risk compared with those in higher resource settings, highlighting the influence of health-system capacity has on trauma outcomes. The aim of this secondary analysis of the GOAL-Trauma dataset was to characterise current variation in pre-operative transfusion practices for patients undergoing trauma laparotomy, and identify any associations between resuscitation strategies and patient mortality.

## Methods

### Study design

This study was a planned secondary analysis of the GOAL-Trauma study, and has been reported according to the STrengthening the Reporting of OBservational studies in Epidemiology (STROBE) guidelines.^23^

The protocol for the GOAL-Trauma study has been published previously.^24^ In summary, patients were recruited between 1st April 2024 and 31st December 2024 during select 30-day periods and followed until discharge, death, or 30 days post-operatively (if still hospitalised), whichever came first. Any healthcare facility worldwide that could perform and manage a patient undergoing a trauma laparotomy was eligible to enrol. Data was collected prospectively by local investigators on all eligible patients throughout pre-selected 30-day period(s); each recruiting centre required a lead investigator (termed the “local lead”), who was asked to complete a site survey to provide further details and contextualise their respective clinical setting.

### Ethics

Ethical approval was granted by the Cambridge Psychology Research Ethics Committee (PRE.2023119) and the study was registered on ClinicalTrials.gov (NCT06180668). Appropriate local approval was obtained by each contributing centre prior to enrolment into the study, according to respective local regulations. In some participating hospitals, informed patient consent was taken, whereas in other hospitals this was deemed not necessary, at the discretion of the local team.

### Participants

Patients of any age who presented with a blunt or penetrating injury and underwent a laparotomy within 5 days (120 h) of presentation to the treating hospital were eligible for inclusion. Patients were excluded if they were undergoing a repeat laparotomy at the recruiting centre (often termed a “relook laparotomy”) within 30 days of the index procedure, or had been recently discharged from any hospital (including for non-trauma related admissions) and had presented within 30 days of discharge.

### Assessments

Blood product usage, including PRBC, FFP, platelets, cryoprecipitate, and whole blood (WB), as well as tranexamic acid (TXA), was recorded between the time of injury and the start of the index procedure. The focus on pre-operative transfusion ensured for an accurate representation of the initial resuscitative response before definitive haemorrhage control, reflecting the distinct clinical phase that may influence early physiological status and subsequent outcomes. In contrast, post-operative transfusion practices were not included, as these reflect any evolving morbidity rather than initial resuscitation requirements.

Injury severity was calculated using the Abbreviated Injury Scale (AIS) for each body region, with the overall degree of injury quantified through the summative Injury Severity Score (ISS).^25^ Patient co-morbidity was quantified through the American Society of Anesthesiologists (ASA) score; this was chosen due to its wide global acceptance and the high level of variation in ability to diagnose select co-morbidities in certain areas. The availability of blood products at each hospital was obtained through a structured reporting tool completed by local investigators at each study site. Hospital- and patient-level data, including pre-operative blood product use and outcome metrics, were uploaded centrally onto a secure web-based system, REDCap cloud,^26^^,^^27^ hosted by the University of Cambridge.

### Outcomes

The primary outcome for the study was 30-day post-operative in-hospital mortality rate.

### Statistical analysis

Patient-level data, including blood product and TXA usage, were reported as descriptive statistics. Contributing hospitals were stratified by their national Human Development Index (HDI),^28^ with HDI being categorised into lower, middle, and upper tertiles. Continuous variables were reported as median with interquartile range (IQR) and compared using the Kruskal–Wallis test, while categorical variables were presented as frequencies and percentages and compared with Pearsons χ^2^ test.

Selection of covariates for the regression model was guided by a directed acyclic graph (DAG), which was determined through a combination of literature review and expert consensus (Supplementary Material 2). This ensured that the regression model accounted appropriately for the causal pathways between the intervention and the outcome, thereby improving the validity and interpretability of the effect estimates. Covariates selected for the model were age, HDI tertile, mechanism of injury, time from injury to operation, ASA score, systolic blood pressure, ISS, estimated blood loss, International Normalised Ratio (INR), and hospital blood product availability. To account for non-linear associations to outcomes, INR and systolic blood pressure were modelled using restricted cubic splines. Multivariable logistic regression was used to identify any association between patients who received pre-operative FFP:PRBC in a ratio ≥1:2 (termed high ratio) versus patients who received pre-operative FFP:PRBC in a ratio <1:2 (termed low ratio), on post-operative 30-day mortality rate; whole blood units were approximated as 1 FFP and 1 RPBC for ratio calculations. Patients who did not receive any form of component therapy or WB, or those with incomplete data variables, were excluded from the regression analysis. A hierarchical model based on hospital was considered, however a significant number of hospitals did not contribute sufficient patient numbers to make such modelling feasible.

Following review of the initial analyses, further exploratory post-hoc multivariable logistic regression analysis was performed with additional adjustment by total volume of transfusion received per patient as a continuous linear variable. The approximate volume of blood products was 1 unit PRBC = 250 mL, 1 unit FFP = 250 mL, 1 pool platelets = 250 mL, and 1 unit whole blood = 500 mL. Separate post-hoc subgroup analysis was performed to assess the unadjusted association between FFP:PRBC ratio and 30-day mortality, across increasing transfusion volume strata; given evidence of non-linearity between volume and mortality observed, volume strata were categorised into increasing 500 mL groups. Formal statistical testing for interaction between transfusion ratio and transfusion volume was not pursued, given the exploratory nature of this analysis and the limited number of patients at higher transfusion volumes. Pre-planned subset analyses were also performed on select groups defined as: [1] high anatomical injury burden defined as any select AIS score of ≥4 from the chest, abdomen, or extremities; [2] physiological derangement defined as a systolic blood pressure <90 mmHg, a serum lactate >6.0 mmol/L, or an International Normalised Ratio (INR) > 1.2; or [3] uncontrolled haemorrhage, defined as intra-operative blood loss >1000 mL.

Variables that had rates of missing data >1% had the missing values imputed using multiple imputation by chained equations. Effect estimates were expressed as odds ratios (OR) with 95% confidence intervals (CI), and p-values <0.05 were considered statistically significant. All analysis was conducted on R (version 4.4.1), using “splines”, “daggity”, “mice” and “dplyr” packages.

### Role of the funding source

The funders had no involvement in study design, data collection, data analyses, data interpretation, or the writing of the report

## Results

### Participant characteristics

The study included a total of 1769 patient records from 187 hospital centres across 51 countries. Among these, 492 patients (27.8%) were from high-HDI settings, 714 (40.4%) from middle-HDI settings, and 563 (31.8%) from low-HDI settings. Complete data on blood product use were available for 1768 patients (99.9%) for inclusion in the final data analysis. Further patient and hospital factors on the cohort can be found in Supplementary Material 3.

### Global trends in blood product and tranexamic acid usage

In the overall cohort, 721 patients (40.8%) received at least one type of blood product in the pre-operative period (Table 1), with the highest proportion observed in the upper HDI tertile (263 patients, 53.5%), compared to those in middle HDI tertile (264 patients, 37.0%) and lower HDI tertile (194 patients, 34.5%) respectively (p < 0.0001).

**Table 1:** Proportion of patients who received the respective blood products and TXA across HDI tertile.

	Lower HDI (n = 562)	Middle HDI (n = 714)	Upper HDI (n = 492)	Total (n = 1768)	p-value
Any blood product, n (%)	194 (34.5)	264 (37.0)	263 (53.5)	721 (40.8)	<0.0001
Tranexamic acid, n (%)	144 (25.6)	133 (18.6)	252 (51.2)	529 (29.9)	<0.0001
PRBC, n (%)	119 (21.1)	235 (32.9)	244 (49.6)	598 (33.8)	<0.0001
Platelets, n (%)	26 (4.6)	30 (4.2)	43 (8.7)	99 (5.6)	0.0016
FFP, n (%)	81 (14.4)	155 (21.7)	140 (28.5)	376 (21.3)	<0.0001
Cryoprecipitate, n (%)	0 (0)	11 (1.5)	21 (4.3)	32 (1.8)	<0.0001
WB, n (%)	89 (15.8)	25 (3.5)	35 (7.1)	149 (8.4)	<0.0001

**Fig. 1:**
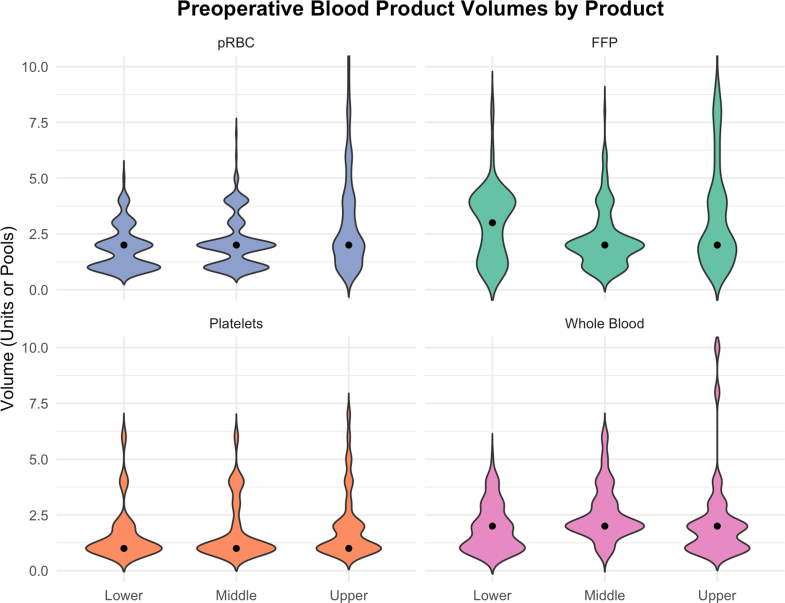
**Violin plot of preoperative blood product volumes across HDI tertiles**. Extreme outliers excluded.

PRBC were the most frequently transfused component across all HDI tertile (598 patients, 33.8%). Their use was highest in the upper HDI tertile (244 patients, 49.6%) compared to the middle tertile (235 patients, 32.9%) and the lower tertile (119 patients, 21.1%) (p < 0.0001). The global median transfusion volume per patient was two units (IQR 1–3), with slightly higher volumes in the upper HDI tertile (p < 0.0001) (Fig. 1). Higher HDI tertile centres were also more likely to use large volumes of WB, FFP and pRBC transfusions compared to middle and lower HDI tertile centres (Fig. 1).

Overall use of FFP globally was 21.3% (376 patients), being more frequently administered in the upper HDI tertile (140 patients, 28.5%), compared to the middle HDI tertile (155 patients, 21.7%) and lower HDI tertile (81 patients, 14.4%), respectively (p < 0.0001). The median volume of FFP transfused per patient was 2 units (IQR 1–4 units) globally. The use of cryoprecipitate specifically was uncommon across all HDI groups, with only 1.8% (32 patients) receiving this, which included no use in the lower HDI tertile group.

Globally, 5.6% of patients (99 patients) received a platelet transfusion, with higher proportions in the upper HDI tertile (43 patients, 8.7%) compared to the middle HDI tertile (30 patients, 4.2%) and lower HDI tertile (26 patients, 4.6%) respectively (p = 0.0016). The median volume of platelets received globally per patient was one pool (IQR 1–2 pools).

**Fig. 2:**
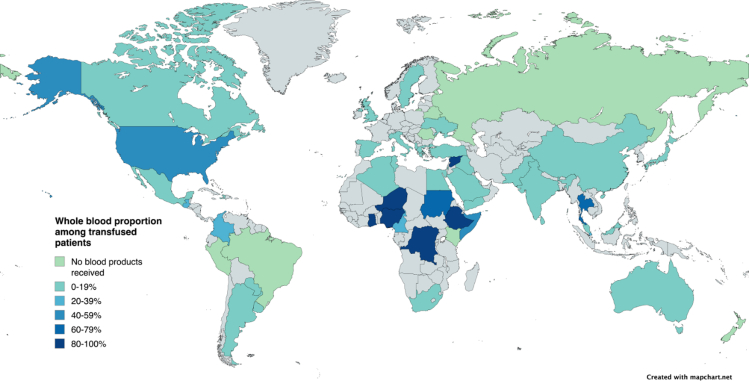
**Global map showing proportion of whole blood use in patients who received any form of blood product (149 of 721 patients)**.

WB was transfused in 149 patients (8.4%) globally. WB usage was significantly higher in lower HDI countries (89 patients, 15.8%, p < 0.0001), compared to the middle HDI tertile (25 patients, 3.5%) and upper HDI tertile (35 patients, 7.1%). The countries with the highest proportion of whole blood transfused patients were predominantly in the lower or middle HDI tertiles (Fig. 2). The median volume of whole blood transfused globally per patient was 2 units (IQR 1–3 units).

The proportion of patients receiving TXA was highest in the upper HDI tertile (252 of 492, 51.2%), compared to the middle HDI (133 of 714, 18.6%) and lower HDI tertile (144 of 562, 25.6%) respectively (p < 0.0001). There was minimal change in the proportions receiving TXA for those with severe injury, uncontrolled haemorrhage, or physiological signs of shock, across all HDI tertiles.

### Global trends in patients not receiving blood products

Overall, 1047 patients did not receive any form of blood product pre-operatively in the study (Table 2). Patients in this cohort had a similar age and sex distribution to the overall cohort, however a higher proportion in the no blood products cohort had suffered a penetrating injury, compared to those who received blood products (62.2% versus 42.0%, p < 0.001). On sub-analysis, the proportions of patients with high anatomical injury burden (25.6% versus 38.7%, p < 0.001), physiological derangement (28.3% versus 52.4%, p < 0.001), or uncontrolled haemorrhage (25.9% versus 54.8%, p < 0.001) were lower in the no blood products cohort, compared to the those who received blood products. The availability of blood products all of the time remained similar across centres between cohorts (65.0% versus 54.8%, p = 0.142). Trends across the HDI tertiles were similar in the no blood product cohort, compared to the overall cohort.

**Table 2:** Patient factors and blood product availability for those who did not receive any blood products, across HDI tertiles.

	Lower HDI (n = 368)	Middle HDI (n = 450)	Upper HDI (n = 229)	Total (n = 1047)	p-value
Age (years), mean (SD)	28.8 (12.4)	34.7 (14.9)	39.9 (16.9)	33.7 (15.1)	<0.0001
Male sex, n (%)	324 (88.0)	403 (89.6)	184 (80.3)	911 (87.0)	0.0025
Mechanism of injury					
Blunt, n (%)	139 (37.8)	149 (33.1)	108 (47.1)	396 (37.8)	0.0020
Penetrating, n (%)	229 (62.2)	301 (66.9)	121 (52.8)	651 (62.2)	
Systolic Blood Pressure on arrival (mmHg), mean (SD)	108 (23)	120 (23)	121 (23)	116 (24)	<0.0001
ISS, median (IQR)	9 (4–16)	9 (4–17)	9 (6–20)	9 (4–16)	<0.0001
Time to operate (hours), mean (SD)	14.8 (21.2)	15.4 (18.3)	15.0 (67.2)	15.1 (35.9)	<0.0001
AIS ≥4 (severe injury), n (%)	81 (22.0)	123 (27.3)	64 (27.9)	268 (25.6)	0.1384
Physiological signs of shock, n (%)	110 (29.9)	132 (29.3)	54 (23.6)	296 (28.3)	<0.0001
Intraoperative blood loss ≥1000 mL, n (%)	92 (25.9) (n = 355)	120 (27.6) (n = 435)	47 (22.5) (n = 209)	259 (25.9) (n = 999)	0.4192
Centre blood product availability					
All of the time	234 (63.6)	237 (52.7)	210 (91.7)	681 (65.0)	<0.0001
Most of the time	125 (34.0)	107 (23.8)	19 (8.3)	251 (24.0)	
Some of the time	9 (2.4)	106 (23.6)	0 (0)	115 (11.0)	

### Blood product usage and mortality outcomes

**Fig. 3:**
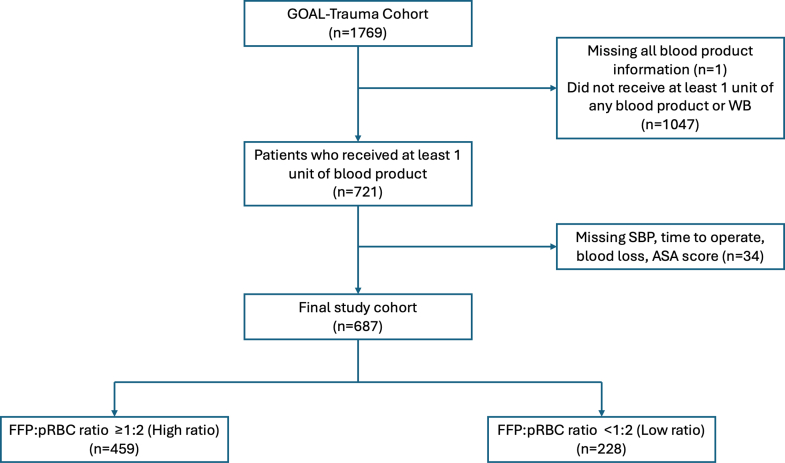
**Cohort selection for high ratio and low ratio of PRBC:FFP usage in trauma laparotomy patients, across 150 hospitals**.

Overall, 721 patients from the overall cohort received at least 1 unit of any blood product (including whole blood), from 150 hospitals. 34 patients were excluded from the analysis due to missing regression variables (estimated blood loss volume, n = 28; time from injury to operation, n = 5; systolic blood pressure, n = 1), resulting in 459 patients in the high ratio group and 228 patients in the low ratio group for analysis (Fig. 3). The distribution of patients across FFP:PRBC ratios is demonstrated in the Supplementary Material 4.

Patients in the FFP:PRBC high ratio group and low ratio group were of similar average age (34.1 years versus 35.5 years, p = 0.509) and sex distribution (83.0% male versus 84.2% male, p = 0.787) (Table 3). There was significant variation in the proportion of patients represented from each HDI tertile (p < 0.0001), with a higher proportion of patients from the lower HDI tertile in the high ratio group compared to the low ratio group (33.3% versus 14.4%), reflecting the higher proportion of WB in these settings. Injury severity scores were similarly high in both groups (median 15 versus 16, p = 0.658), however time to operation was significantly longer in the high ratio group (16.6 h versus 11.4 h, p = 0.029). Patients receiving high ratio of FFP:PRBC had a higher median ASA score compared to those in the low ratio group (3 versus 2, p = 0.005). There was a high amount of missing data for INR in the overall cohort (243 of 687 patients, 35.4%); missing INR values were imputed using multivariable multiple imputation by chained equations, with predictive models incorporating ISS, systolic blood pressure, Glasgow Coma Score, heart rate, age, time to presentation, mechanism of injury, and ASA grade. After imputation, admission INR was similar in both cohorts (1.29 versus 1.26, p = 0.620).

**Table 3:** Patient factors and blood product availability, between high ratio and low ratio cohorts.

	High ratio cohort (n = 459)	Low ratio cohort (n = 228)	Total (n = 687)	p-value
Age (years), mean (SD)	34.1 (16.4)	35.5 (17.1)	34.5 (16.6)	0.509
Male sex, n (%)	381 (83.0)	192 (84.2)	573 (83.4)	0.787
Mechanism of injury				
Blunt, n (%)	273 (59.5)	128 (56.1)	401 (58.4)	0.559
Penetrating, n (%)	186 (40.5)	100 (43.9)	286 (41.6)	
Systolic Blood Pressure on arrival (mmHg), mean (SD)	98 (30)	103 (29)	100 (30)	0.057
ISS, median (IQR)	15 (9–25)	16 (9–25)	16 (9–25)	0.658
Time to operation (hours), mean (SD)	16.6 (70.1)	11.4 (19.4)	14.9 (58.4)	0.029
AIS ≥4 (severe injury), n (%)	174 (37.9)	94 (41.2)	268 (39.0)	0.449
Physiological signs of shock, n (%)	252 (54.9)	110 (48.2)	362 (52.7)	0.052
Intraoperative blood loss >1000 mL, n (%)	238 (51.9)	132 (57.9)	370 (53.9)	0.228
Centre blood product availability				
All of the time	297 (64.7)	174 (76.3)	471 (68.6)	0.006
Most of the time	142 (30.9)	45 (19.7)	187 (27.2)	
Some of the time	20 (4.4)	9 (3.9)	29 (4.2)	

**Fig. 4:**
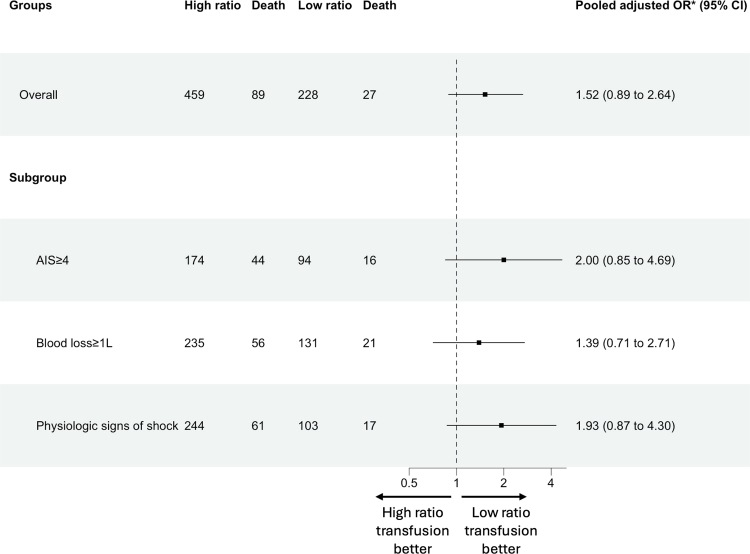
**Forest plot (log scale) of multivariate analysis for 30-day mortality, comparing high ratio and low ratio cohorts, including subgroup analysis**.

The 6-h post-operative crude mortality rates in the high ratio group was 5.2% (24 of 459 patients) and 2.63% in the low ratio group (6 of 228 patients). The 24-h post-operative crude mortality rates in the high ratio group was 8.5% (39 of 459 patients) and 5.7% in the low ratio group (13 of 228 patients). The 30-day post-operative crude mortality rate in the high ratio group was 19.4% (89 of 459 patients) and 11.8% in the low ratio group (27 of 228 patients). Following multivariate regression analysis, no difference in 30-day post-operative mortality was observed between the high ratio and low ratio group (OR = 1.52, CI: 0.89–2.64) (Fig. 4). Separate multivariate analysis with the addition of blood product transfusion volume as a co-variate showed similar outcomes (Supplementary Material 5). A separate DAG-informed adjustment set was identified, comprising the minimally sufficient variables required to block backdoor confounding pathways, with similar outcomes also observed (OR = 1.49, CI: 0.91–2.42). At sub-group analysis, no significant differences between the two cohorts were observed for those with high anatomical injury burden (OR = 2.00, CI: 0.85–4.69), physiological derangement (OR = 1.93, CI: 0.87–4.30), or uncontrolled haemorrhage (OR = 1.39, CI: 0.71–2.71).

**Fig. 5:**
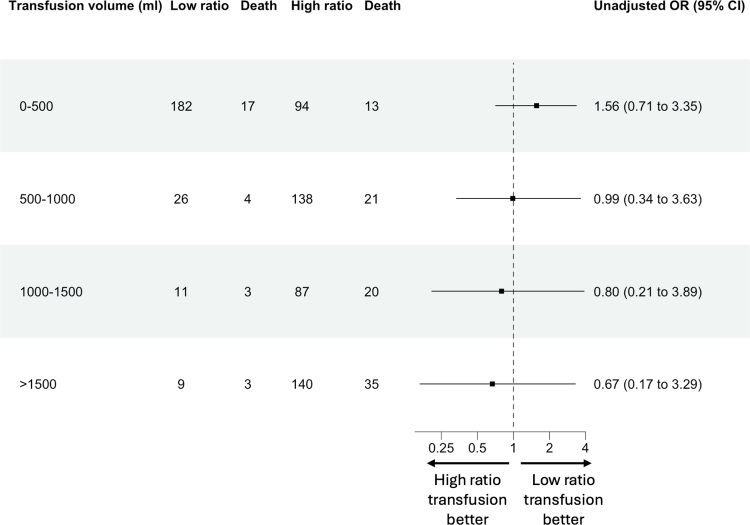
**Forest plot (log scale) of 30-day mortality, comparing high ratio and low ratio cohorts, across increasing transfusion volume**.

The median volume transfused in the low ratio group was 2 units and in the high ratio group was 4 units. Across the cohort, crude mortality rate increased with overall transfusion volume (OR 1.10 per unit transfused, CI: 1.06–1.14; p < 0.0001). Unadjusted analysis of mortality risk between the high ratio and low ratio cohorts showed no significant definitive differences in outcome with increasing volume of transfusion (Fig. 5).

## Discussion

This is the first study to map the global patterns of blood product use in trauma patients, reporting transfusion practices from 1768 patients across 51 countries. We have demonstrated clear trends towards higher blood product usage overall in the higher resource settings, however there was a notably greater use of whole blood in lower resource settings. While no difference was observed in mortality risk between high ratio and low ratio of FFP to PRBC in the overall cohort, we did note a trend towards improved outcomes for those patients receiving higher volumes of transfusion in the high ratio group. This study provides essential data to the ongoing discussion regarding optimal blood transfusion practices globally and emphasises the need for reciprocal learning across settings, especially in the use of whole blood in trauma.

Approximately twice the number of patients proportionately received blood products pre-operatively in the upper HDI setting, compared to those in the lower HDI setting, across each of PRBC, platelets, and FFP. These disparities likely reflect the prerequisites necessary for component separation and storage, with the substantial financial and infrastructural requirements often not achievable or consistently available in lower-resource settings. Indeed, among blood donations globally, only 38% of donations in low-income countries are separated into components, compared to the 96% in high-income countries.^29^ Access to appropriate blood products in lower resource settings is known to be challenging, as previous reports have demonstrated only around 6–16% of individuals in LMICs receive any form of blood product following a traumatic injury.^30^ Our data echoes this; for patients with physiological signs of shock following injury, a cohort who are arguably likely to require blood product resuscitation, a higher proportion of patients from lower and middle HDI tertiles did not receive any form of blood products, compared to those in upper HDI tertile. This further supports current literature on “blood gap” in low-resource settings^31^; with over half of countries worldwide unable to provide sufficient blood products to meet their population demand,^32^ the development of any global consensus guidance for the use of blood products in trauma resuscitation must ensure such considerations are accounted for.

In this context, there is an increasing interest for the use of WB in trauma resuscitation. Recent evidence has suggests that WB may provide a more comprehensive volume and coagulation factor replacement compared to any component therapy when transfused early.^18^, ^19^, ^20^^,^^33^ Arguably more important on the global stage is the relative ease with which WB can be processed and stored, with a much lower facility-level infrastructure demand.^29^ There is little evidence published regarding the proportion of trauma patients receiving WB in lower resource settings, however our data demonstrated significantly higher rates of whole blood usage across the lower HDI tertile, with over double the proportion of patients receiving whole blood compared to those treated in middle and upper HDI tertiles. While much of the formal published research into WB use in trauma is derived from higher-resource nations and military use, the use of WB as a primary blood product in trauma has been the norm for decades in many lower-resource settings. As a result, these centres have accumulated extensive practical experience in donor mobilisation and transfusion pathways. With the recent resurgence of interest in WB in high-resource nations, these centres offer valuable operational insights that may help optimise the reintroduction of WB programmes in higher-resource countries. Ensuring a bidirectional dialogue and encouraging reverse innovation is key for the experiences and insights of WB use to be implemented for the wider global practice.

Overall, tranexamic acid was used in less than a third of cases globally, albeit use rising to around half of cases in the upper HDI tertile. Tranexamic acid has a strong evidence base for use in trauma, with both consensus-guidelines and clinical trials, such as the CRASH-2 study, recommending its use for all patients with known or suspected bleeding,^13^^,^^34^ especially if given within 3 h of injury.^35^ With an overall low relative cost and long shelf-life, the limited utilisation we observed with its use, especially in the lower resource settings, needs addressing. At present, however, reasoning for this remains mainly conjecture. Possible rationale includes the often prolonged delays patients face prior to presentation to a hospital, meaning where tranexamic acid would have limited clinical benefit,^36^ or more procurement and supply-related reasons. Further research on this area is now required.

Of interest, our cohort showed that a majority of patients (1047 of 1768 patients) who underwent trauma laparotomy did not receive any pre-operative blood products. There are several factors that may have contributed to this finding. Firstly, a vast majority of trauma patients do not require transfusion, with the literature suggesting that only 15–20% of trauma patients require blood transfusion and only 3% of patients require massive transfusions.^37^^,^^38^ A majority of the cohort may not have had physiological evidence of major haemorrhage or may have sustained injuries that did not demonstrate ongoing bleeding. Secondly, variability in access to blood products, particularly in low resource settings, may have contributed to limited availability, procurement delays, or storage constraints reducing the likelihood of receiving transfusion even when clinically indicated. This was supported by our data, which showed that majority of patients who did not receive blood products prior to laparotomy were from low and middle HDI countries (818 out of 1047 patients). Together, these factors highlight the complexity and heterogeneity of early resuscitation pathways in trauma across diverse global healthcare settings.

In an attempt to assess optimal blood product resuscitation strategy from a global cohort, we compared low ratio to high ratio of FFP to PRBC, with all patients who received WB comprised within the high ratio group. While no improvement to mortality was observed in either group when analysing across the whole cohort, at higher blood transfusion requirements there was a suggestion of improved mortality outcomes in the high ratio cohort; benefits of a high ratio of FFP to PRBC is in keeping with the current wider literature,^39^ however in this study no definitive association between transfusion volume and mortality could be established, likely due to limited sample size at higher transfusion volumes. Importantly, this study is the first global study to also include WB in such analysis and provides important real-world data to add to the discussion on optimal blood transfusion practices in trauma. Taking such conclusions into consideration when devising future studies and drafting any global trauma resuscitation guidelines is therefore crucial. Indeed, any beneficial role WB can have globally must be used to assist in the design of future randomised trials, specifically that inclusion criteria for a trial of WB usage might be restricted to patients who are predicted to have a higher transfusion requirement, or that stratified randomisation and prespecified subgroup analyses should include predicted transfusion requirement. This is an essential area of future work in global blood transfusion strategy.

We have reported data from a large international multi-centre prospective observational study, providing key insights into current transfusion practices globally. However, this work does come with several limitations that impact how this work should be interpreted. As an observational study, this work is at risk of unaccounted confounding factors. We have attempted to mitigate this through the use of a consensus-derived and evidence-based DAG prior to our analysis. Furthermore, this is a secondary analysis, with the primary study not being specifically powered for this analysis, meaning a greater chance of type 2 error. As a result we have been cautious not to over-interpret our study findings. Crucially, our statistical analysis does not account for the temporal sequence of transfusion, nor blood products given intra-operatively or post-operatively, which are evidently important factors within blood transfusion practices. In addition, for ratio calculations we approximated whole blood as equivalent to one unit of RBC and one unit of FFP; this assumption may not fully capture the advantages of whole blood over individual blood components and may introduce imprecision into ratio-based comparisons. The majority of participating hospitals in the study were urban tertiary hospitals, which limits the implications to more rural primary hospital settings. Our transfusion volume sub-analysis has been performed on the assumption of a linear relationship to outcome metrics, whilst the true relationship may be non-linear; whilst we have attempted to adjust for this using volume subgroup assessment, further work is still required in this area to explore the relationship between volume and outcome. Finally, our analysis was restricted to patients undergoing trauma laparotomy, and our findings may not be fully generalisable to all trauma patients. However, the substantial variation in transfusion practices observed even within this single, common emergency surgical procedure highlights the complexity and heterogeneity of global trauma resuscitation.

In conclusion, this secondary post-hoc analysis of the GOAL-Trauma study found substantial variations in pre-operative blood product resuscitation practices among trauma laparotomy patients across different HDI settings. Patients from the upper HDI tertile received a higher overall use of blood product components, whereas whole blood transfusions were more prevalent in the lower HDI tertile. Whilst no differences in mortality rates were observed when varying FFP to PRBC ratios in the overall cohort, there was an apparent trend towards improved outcomes in patients requiring higher volume transfusions in the higher FFP to PRBC patient cohort, which included whole blood use. Further work is now required to investigate the role of whole blood in the context of global trauma resuscitation, ensuring that cultural, financial, and resource factors are considered before any guidance is produced, to ensure optimal patient outcomes can be achieved worldwide.

## Contributors

MFB, KK, ZBP, and TB: study design, data collection, data analysis, data interpretation, and writing. JA and TCH: study design, data collection, data interpretation, and writing. LH, BGS, RM, and TGW: study design, data analysis, data interpretation, and writing. WHN: data analysis, data interpretation, and writing. DUB, EC, CMNG: data collection, data interpretation, and writing. All authors had full access to all the data in the study and had final responsibility for the decision to submit for publication. WHN, MFB, KK, and TB accessed and verified the underlying data.

## Data sharing statement

Data is available to any researcher who provides a methodologically sound study proposal that is approved by the Global Outcomes After Laparotomy for Trauma (GOAL-Trauma) steering committee. Proposals may be submitted to the International Health Systems Group at the University of Cambridge. Individual patient or hospital data will not be identifiable in any released data and all appropriate information governance protocols will be followed.

## Editor note

The Lancet Group takes a neutral position with respect to territorial claims in published maps and institutional affiliations.

## Declaration of interests

MFB is funded by the Royal College of Surgeons of England Ratanji Dalal Research Fellowship and the Engineering and Physical Sciences Research Council (EPSRC). WHN was supported by the Royal College of Physicians Wolfson Intercalated Award. LH and TB are funded by the NIHR (ref: NIHR132455) using UK aid from the UK Government to support global health research.

## References

[1] World Health Organization (2021). Injuries and violence fact sheet. https://www.who.int/news-room/fact-sheets/detail/injuries-and-violence.

[2] Mock C., Joshipura M., Arreola-Risa C., Quansah R. (2012). An estimate of the number of lives that could be saved through improvements in trauma care globally. World J Surg.

[3] Higashi H., Barendregt J.J., Kassebaum N.J., Weiser T.G., Bickler S.W., Vos T. (2015). Burden of injuries avertable by a basic surgical package in low- and middle-income regions: a systematic analysis from the Global Burden of Disease 2010 Study. World J Surg.

[4] Bashford T., Clarkson P.J., Menon D.K., Hutchinson P.J.A. (2018). Unpicking the Gordian knot: a systems approach to traumatic brain injury care in low-income and middle-income countries. BMJ Glob Health.

[5] Hardcastle T.C., Gaarder C., Balogh Z. (2025). Guidelines for Enhanced Recovery After Trauma and Intensive Care (ERATIC): Enhanced Recovery After Surgery (ERAS) and International Association for Trauma Surgery and Intensive care (IATSIC) Society recommendations: part 3: trauma ethics and systems aspects. World J Surg.

[6] Wiik Larsen J., Søreide K., Søreide J.A., Tjosevik K., Kvaløy J.T., Thorsen K. (2022). Epidemiology of abdominal trauma: an age- and sex-adjusted incidence analysis with mortality patterns. Injury.

[7] Jones E.L., Stovall R.T., Jones T.S. (2014). Intra-abdominal injury following blunt trauma becomes clinically apparent within 9 hours. J Trauma Acute Care Surg.

[8] Arumugam S., Al-Hassani A., El-Menyar A. (2015). Frequency, causes and pattern of abdominal trauma: a 4-year descriptive analysis. J Emerg Trauma Shock.

[9] Harvin J.A., Maxim T., Inaba K. (2017). Mortality after emergent trauma laparotomy. J Trauma Acute Care Surg.

[10] Bath M.F., Bashford T., GOAL-Trauma Collaborative (2024). GOAL-Trauma Collaborative. The trauma laparotomy-A key procedure that lacks global data. World J Surg.

[11] Bath M.F., Schloer J., Strobel J. (2024). Trends in pre-hospital volume resuscitation of blunt trauma patients: a 15-year analysis of the British (TARN) and German (TraumaRegister DGU®) National Registries. Crit Care.

[12] Moore E.E., Moore H.B., Kornblith L.Z. (2021). Trauma-induced coagulopathy. Nat Rev Dis Primers.

[13] Roberts I., Bautista R., CRASH-2 trial collaborators (2010). Effects of tranexamic acid on death, vascular occlusive events, and blood transfusion in trauma patients with significant haemorrhage (CRASH-2): a randomised, placebo-controlled trial. Lancet.

[14] World Health Organization (2025). https://www.who.int/publications/i/item/B09474.

[15] Holcomb J.B., del Junco D.J., Fox E.E. (2013). The prospective, observational, multicenter, major trauma transfusion (PROMMTT) Study. JAMA Surg.

[16] Holcomb J.B., Tilley B.C., Baraniuk S. (2015). Transfusion of plasma, platelets, and red blood cells in a 1:1:1 vs a 1:1:2 ratio and mortality in patients with severe trauma. JAMA.

[17] Mohammed A.D., Ntambwe P., Crawford A.M. (2020). Barriers to effective transfusion practices in limited-resource settings: from infrastructure to cultural beliefs. World J Surg.

[18] Hazelton J.P., Ssentongo A.E., Oh J.S. (2022). Use of cold-stored whole blood is associated with improved mortality in hemostatic resuscitation of major bleeding. Ann Surg.

[19] van der Horst R.A., Rijnhout T.W.H., Noorman F. (2023). Whole blood transfusion in the treatment of acute hemorrhage, a systematic review and meta-analysis. J Trauma Acute Care Surg.

[20] Meizoso J.P., Cotton B.A., Lawless R.A. (2024). Whole blood resuscitation for injured patients requiring transfusion: a systematic review, meta-analysis, and practice management guideline from the Eastern Association for the Surgery of Trauma. J Trauma Acute Care Surg.

[21] Ripoll-Gallardo A., Caviglia M., Ratti M. (2024). Fresh whole blood: a feasible alternative in disasters and mass casualty incidents? A systematic review and meta-analysis. Confl Health.

[22] Bath M.F., Amoako J., Kohler K. (2025). Global variation in patient factors, interventions, and postoperative outcomes for those undergoing trauma laparotomy: an international, prospective, observational cohort study. Lancet Glob Health.

[23] von Elm E., Altman D.G., Egger M., Pocock S.J., Gøtzsche P.C., Vandenbroucke J.P. (2008). STROBE Initiative. The strengthening the reporting of Observational Studies in Epidemiology (STROBE) statement: guidelines for reporting observational studies. J Clin Epidemiol.

[24] Bath M.F., Kohler K., Hobbs L. (2024). Evaluating patient factors, operative management and postoperative outcomes in trauma laparotomy patients worldwide: a protocol for a global observational multicentre trauma study. BMJ Open.

[25] Gennarelli T., Woodzin E., Association for the Advancement of Automotive Medicine (2016). Abbreviated Injury Scale (c) 2005 Update 2008.

[26] Harris P.A., Taylor R., Minor B.L. (2019). The REDCap consortium: building an international community of software platform partners. J Biomed Inf.

[27] Harris P.A., Taylor R., Thielke R., Payne J., Gonzalez N., Conde J.G. (2009). Research electronic data capture (REDCap)—A metadata-driven methodology and workflow process for providing translational research informatics support. J Biomed Inf.

[28] United Nations Development Programme (2025). Human development data. https://hdr.undp.org/data-center.

[29] World Health Organization (2025). https://www.who.int/news-room/fact-sheets/detail/blood-safety-and-availability.

[30] Yang L., Slate-Romano J., Marqués C.G. (2020). Evaluation of blood product transfusion therapies in acute injury care in low- and middle-income countries: a systematic review. Injury.

[31] Kralievits K.E., Raykar N.P., Greenberg S.L.M., Meara J.G. (2015). The global blood supply: a literature review. Lancet.

[32] Roberts N., James S., Delaney M., Fitzmaurice C. (2019). The global need and availability of blood products: a modelling study. Lancet Haematol.

[33] Brill J.B., Tang B., Hatton G. (2022). Impact of incorporating whole blood into hemorrhagic shock resuscitation: analysis of 1,377 consecutive trauma patients receiving emergency-release uncrossmatched blood products. J Am Coll Surg.

[34] Rossaint R., Afshari A., Bouillon B. (2023). The European guideline on management of major bleeding and coagulopathy following trauma: sixth edition. Crit Care.

[35] Roberts I., Shakur H., CRASH-2 collaborators (2011). The importance of early treatment with tranexamic acid in bleeding trauma patients: an exploratory analysis of the CRASH-2 randomised controlled trial. Lancet.

[36] Wogu A.F., Dixon J.M., Xiao M. (2025). Tranexamic acid is associated with post-injury mortality in a resource-limited trauma system: findings from the epidemiology and outcomes of prolonged trauma care cohort study. Transfusion (Paris).

[37] Sisak K., Manolis M., Hardy B.M., Enninghorst N., Bendinelli C., Balogh Z.J. (2013). Acute transfusion practice during trauma resuscitation: who, when, where and why?. Injury.

[38] Como J.J., Dutton R.P., Scalea T.M., Edelman B.B., Hess J.R. (2004). Blood transfusion rates in the care of acute trauma. Transfusion (Paris).

[39] Hynes A.M., Geng Z., Schmulevich D. (2021). Staying on target: maintaining a balanced resuscitation during damage-control resuscitation improves survival. J Trauma Acute Care Surg.

