# Supplementary material for: Use of pre-operative blood products in abdominal trauma: a planned secondary analysis of the GOAL-trauma study
Source: eClinicalMedicine. 2026 Mar 4;93:103814. doi: 10.1016/j.eclinm.2026.103814 (PMC13043316; doi:10.1016/j.eclinm.2026.103814)
Supplement: Supplementary Material [file mmc1.docx]

# Supplementary Material

1 – List of collaborators

2 – Directed Acyclic Graph

3 – Patient factors and blood product availability across the entire cohort

4 – Histogram of patient FFP:PRBC ratio across the entire cohort

5 – Forest plot of multivariate analysis for 30-day mortality, comparing high ratio and low ratio cohorts, with the addition of blood product transfusion volume as a co-variate

**Supplementary Material 1 - List of Collaborators**

***Writing Group***

WH Ng, MF Bath, J Amoako, K Kohler, DU Baderhabusha, EL Caceres, CM Nuño-Guzmán, L Hobbs, BG Smith, TG Weiser, TC Hardcastle, ZB Perkins, T Bashford

***Protocol Development Group***

MF Bath, T Edmiston, BG Smith, D Clarke, A Kwizera, L Hobbs, K Kohler, A Mazzoleni, FFI Fareed, Z Zhang, R Thavayogan, J Erhabor, O Mantle, C Hammer, Z Perkins, M Marsden, R Davenport, RJ Davies, J Amoako, R Moonesinghe, TG Weiser, A Leather, TC Hardcastle, R Naidoo, YRA Nordín Servín, A Conway Morris, K Lakhoo, GA Bass, JM Wohlgemut, P Hutchinson, T Bashford

***Statistical Analysis Team***

WH Ng, MF Bath, K Kohler, ZB Perkins

***Hospital Leads***

*Albania*: A Dogjani (University Trauma Hospital; Tirana)

*Algeria*: A Tidjane (EHU-1st November 1954; Oran)

*Argentina*: SE Vélez (Hospital de Urgencias de Córdoba; Córdoba); J Lopez (Hospital de Emergencias Dr Clemente Alvarez; Rosario)

*Australia*: C O’Flynn (The Alfred Hospital; Melbourne)

*Bahrain*: F Haider (Salmaniya Medical Complex Government Hospital; Manama)

*Belarus*: A Litvin (Gomel State Medical University; Gomel)

*Brazil*: R Filho (Hospital Regional Dom Moura; Garanhuns)

*Cameroon*: T Tientcheu (Yaoundé Central Hospital; Yaoundé); S Eya (Centre des Urgence de Yaoundé; Yaoundé)

*Canada*: EG Wong (McGill University Health Centre; Montreal)

*China*: R Wang (Shanghai General Hospital, Shanghai Jiao Tong University School of Medicine, Shanghai); J Wang (Yantai Yuhuangding Hospital, Affiliated with Medical College of Qingdao University; Yantai); Y Ni (The Affiliated Changzhou No.2 People's Hospital of Nanjing Medical University; Changzhou); Z Wang (The People's Hospital of Yuyao City, The Affiliated Yangming Hospital of Ningbo University; Ningbo); Z Tian (Beijing Jishuitan Hospital, Capital Medical University; Beijing); M Fang (Hebei Medical University Third Hospital; Shijiazhuang); M Zhou (The Second Affiliated Hospital of Zhengzhou University; Zhengzhou); S Liu (Zhongda Hospital, Southeast University; Nanjing); K Xie (Tianjin Medical University General Hospital; Tianjin); Z Zhang (Sir Run Run Shaw Hospital, Zhejiang University School of Medicine; Hangzhou); X Guo (Dongguan Eastern Central Hospital, The Sixth Affiliated Hospital of Jinan University; Dongguan); Y Ke (The Second Affiliated Hospital of Kunming Medical University; Kunming); H Ni (Affiliated Jinhua Hospital, Zhejiang University School of Medicine; Jinhua); Q Luo (Shanghai Pudong New Area Gongli Hospital; Shanghai)

*Colombia*: E Caceres (Clínica Universidad de la Sabana; Chía) and LF Reyes (Clínica Universidad de la Sabana; Chía); L Pino (Hospital Universitario del Valle; Cali)

*Democratic Republic of the Congo*: DU Baderhabusha (Hôpital de Kyeshero; Goma); B Cissa Wa Numbe (Hôpital Provincial Général de Référence de Bukavu; Bukavu)

*Egypt*: A Meselhi (Al-Ahrar Zagazig Teaching Hospital; Zagazig); MM Elsayed (Mansoura Specialized Hospital; Mansoura; Delta Hospital; Mansoura); S Abdelmohsen (Aswan University Hospital; Aswan); A Elkhouly (Tanta University Hospital; Tanta); A Elmorshdy (Mataria Teaching Hospital; Cairo); G Abouelnagah (Alexandria Main University Hospital; Alexandria); A Osman (Beni Suef Health Insurance Hospital; Beni Suef); H Taher (Kasr Al Ainy Hospital; Cairo)

*Ethiopia*: W Shenkutie (Arsi University Asella Referral and Teaching Hospital; Asella); M Senbu (Adama Hospital Medical college; Adama City); N Bayleyegn (Jimma University Medical Centre; Jimma); M Merene (ALERT Comprehensive Specialized Hospital; Addis Ababa); M Ahmed (St. Paul’s Hospital Millenium Medical College; Addis Ababa)

*Ghana*: S Gudugbe (Holy Family Catholic Hospital; Techiman); J Amoako (Korle Bu Teaching Hospital; Accra); M Morna (Cape Coast Teaching Hospital; Cape Coast); E Gyabaah (Sunyani Teaching Hospital; Sunyani); H Seidu-Aroza (Ho Teaching Hospital; Ho)

*Greece*: I Baloyiannis (General University Hospital of Larissa; Larissa); K Bouchagier (University Hospital of Patras; Patras); F Mulita (General Hospital of Aigio; Aigio); A Ioannidis (AHEPA University Hospital of Thessaloniki; Thessaloniki)

*Guatemala*: M Rivera (Hospital General San Juan de Dios; Guatemala City)

*Haiti*: F Régis (Hôpital Universitaire La Paix; Port-au-Prince)

*India*: L Bains (Maulana Azad Medical College; New Delhi); M Khajanchi (Seth GS Medical College and KEM Hospital; Mumbai)

*Italy*: L Sartarelli (Presidio Ospedaliero Centrale - SS. Annunziata; Taranto); R Bollino (Azienda USL - IRCCS di Reggio Emilia; Reggio Emilia); M Fedi (San Jacopo Hospital, Pistoia); A Bottari (Ospedale S. Maria alla Gruccia; Montevarchi); F Cammelli (Azienda Ospedaliero Universitaria Careggi; Firenze); G Calini (IRCCS Azienda Ospedaliero - Universitaria di Bologna; Bologna); A Piccolo (Grande Ospedale Metropolitano; Reggio Calabria); D Visconti (AOU Cittá della Salute e della Scienza; Torino); M Altomare (ASST Grande Ospedale Metropolitano Niguarda; Milan); L Carenzo (IRCCS Istituto Clinico Humanitas; Milan); F Fleres (AOU Policlinico G Martino; Messina)

*Japan*: Y Iwao (Ohta Nishinouchi Hospital; Fukushima)

*Kenya*: R Parker (Tenwek Hospital; Tenwek)

*Malaysia*: CK Tiong (Universiti Malaya Medical Centre; Kuala Lumpur); CX Teoh (Hospital Canselor Tuanku Muhriz UKM; Kuala Lumpur); AD Zakaria (USM Specialist Hospital, Universiti Sains Malaysia; Kubang Kerian)

*Mexico*: CM Nuño-Guzmán (Hospital Civil de Guadalajara Fray Antonio Alcalde; Guadalajara); A González-Ojeda (Centro Médico Nacional de Occidente IMSS; Guadalajara)

*New Zealand*: C Wakeman (Christchurch Hospital; Christchurch)

*Niger*: E Ikwutah (SIM Galmi Hospital; Galmi)

*Nigeria*: M Daniyan (Ahmadu Bello University Teaching Hospital; Zaria); A Adamu (Abubakar Tafawa Balewa University Teaching Hospital; Bauchi); E Akpo (Delta State University Teaching Hospital; Oghara); I Chukwu (Federal Medical Centre; Umuahia); M Bashiru (Federal Medical Center Nguru; Nguru); B Akanni (Alex Ekwueme Federal University Teaching Hospital; Abakaliki); J Olaogun (Ekiti State University Teaching Hospital; Ado Ekiti); B Nomayo-Oriabure (The Hills Medical Center; Benin City); H Abiyere (Federal Teaching Hospital Ido-Ekiti; Ido Ekiti); E Oriabure (University of Benin Teaching Hospital; Benin City)

*Pakistan*: FF Khidri (Liaquat University of Medical and Health Sciences; Jamshoro); SA Naqi (Indus Hospital and Health Network; Karachi); T Khan (Lady Reading Hospital; Peshawar); K Faheem (PAF Hospital Base Faisal; Karachi)

*Paraguay*: RS Pederzoli (Hospital de Trauma Prof. Dr. Manuel Giagni; Asunción)

*Occupied Palestinian Territories*: H Abu-Arish (Al-Ahli Hospital and Hebron Governmental Hospital; West Bank); M Youssef (Nasser Hospital; Gaza)

*Peru*: C Huaroto-Landeo (Clinica Internacional, Lima)

*Portugal*: I Carolino Gomes (Unidade Local de Saúde de Lisboa Ocidental; Lisboa); N Gatta (Unidade Local de Saúde São João; Porto)

*Romania*: I Negoi (Clinical Emergency Hospital of Bucharest; Bucharest)

*Russia*: S Katorkin (Clinics of Samar Medical University; Samara)

*Saudi Arabia*: N Alsubaie (King Saud University Medical City; Riyadh)

*Singapore*: JTT Goo (Khoo Teck Puat Hospital; Singapore); S Balasubramaniam (Tan Tock Seng Hospital; Singapore)

*Somalia*: MS Hassan (Mogadişu Somali-Türkiye Recep Tayyip Erdoğan Training and Research Hospital; Mogadishu); SA Mohamed (Kakaal Hospital; Mogadishu); AE Abdishakur (Somali-Sudanese Specialized Hospital; Mogadishu)

*South Africa*: TC Hardcastle (Inkosi Albert Luthuli Central Hospital; Durban); R Naidoo (Ngwelezana Hospital; Empangeni); R Crawford (Chris Hani Baragwanath Academic Hospital; Johannesburg); M Moeng (Charlotte Maxeke Johannesburg Academic Hospital; Johannesburg); HJ Kruger (Tygerberg Hospital; Cape Town)

*Spain*: M Serrano-Navidad (Hospital General Universitario de Elche; Alicante); A Landaluce-Olavarria (Urduliz Hospital; Bizkaia); CC Lopes Moreira (Hospital Universitario Donostia; San Sebastián); H Llaquet-Bayo (Hospital Universitari Parc Taulí; Sabadell)

*Sri Lanka*: K Jayasuriya (District General Hospital Kegalle; Kegalle); JASB Jayasundara (District General Hospital; Dambulla); D Subasinghe (National Hospital of Sri Lanka; Colombo); J Mithushan (Teaching Hospital Batticaloa; Batticaloa)

*Sudan*: A Ibrahim (Atbara Teaching Hospital; Atbara); M Elnour (Prince Digna Referral Hospital; Port Sudan); I Ahmed (Al Hasahissa Teaching Hospital; Al-Hasahisa); I Adel (Bashair Teaching Hospital; Khartoum); L Mohammed (Port Sudan Teaching Hospital; Port Sudan); S Bakhit (Dongola Specialized Hospital; Dongola); M Elbashier (Kassala Teaching Hospital; Kassala City); R Musa (Alnao Teaching Hospital; Omdurman City); J Amin (Sinnar Teaching Hospital; Sinnar City); M Yassin (Gadarif Teaching Hospital; Gadarif); A Babiker (Ad-Damazin Teaching Hospital; Ad-Damazin); A Noureldin (New Halfa Teaching Hospital; New Halfa); A Abdalazeez (Zalingei Teaching Hospital; Zalingei City)

*Sweden*: S Benediktsdottir (Skåne Universitetssjukhus Malmö; Malmö)

*Syria*: L Hasan (Damascus Hospital; Damascus); S Hamad (Al Mouwasat Hospital; Damascus); N Mansour (Homs University Hospital; Homs)

*Thailand*: O Homchan (Maharaj Nakorn Chiang Mai Hospital; Chiang Mai)

*Tunisia*: A Hasnaoui (Menzel Bourguiba Hospital; Bizerte); A Bouzid (Mahmoud Matri Hospital; Ariana); W Riahi (Beja Hospital; Beja)

*Türkiye*: M Ergenç (Marmara University School of Medicine; Istanbul); B Yigit (Bagcilar Training and Research Hospital; Istanbul); B Citgez (Uskudar University Faculty of Medicine, Memorial Hospital; Istanbul); M Yilmaz (Kocaeli City Hospital; Izmit); YF Aydoğdu (Bandirma Training and Research Hospital; Balıkesir); A Guner (Karadeniz Technical University Faculty of Medicine, Farabi Hospital; Trabzon); H Karakullukcu (Sultan Abdülhamid II Han Training and Research Hospital; Istanbul); K Tuncer (Bakircay University Cigli Education and Research Hospital; Izmir); AN Sanli (Private ADN International Hospital; Gaziantep); MT Demirpolat (University of Health Sciences Umraniye Training and Research Hospital; Istanbul); Ç Büyükkasap (Gazi University Hospital; Ankara); AC Yildirim (Kütahya City Hospital; Kütahya); F Feratoglu (Sultanbeyli State Hospital; Istanbul)

*Ukraine*: S Smoliar (Kharkiv Regional Clinical Hospital; Kharkiv)

*United Kingdom*: H Roocroft (Southmead Hospital; Bristol); G McKnight (University Hospital of Wales, Cardiff); M Hughes (Royal London Hospital; London); JV Taylor (University Hospital Aintree; Liverpool); E Yung (Aberdeen Royal Infirmary; Aberdeen); EJ Nevins (Sunderland Royal Hospital; Sunderland); S Owen-Smith (University Hospital Plymouth; Plymouth); A Mian (John Radcliffe Hospital; Oxford); M Alfa-Wali (St Mary’s Hospital; London); C Menichetti (Queen Elizabeth Hospital; Birmingham); T Jodlowski (Salford Royal Hospital; Salford); S Mundell (Morriston Hospital; Swansea); O Khalil (Norfolk and Norwich University Hospital; Norwich); S Jay (Addenbrooke’s Hospital; Cambridge); M El-Boghdady (St George’s University Hospital; London); P Pratheepan (North Middlesex University Hospital; London); A Abouelnaga (Manchester Royal Infirmary; Manchester); A Brooks (Nottingham University Hospitals; Nottingham); S Yoong (Royal Victoria Hospital; Belfast); Y Al Azzawi (Royal Infirmary of Edinburgh; Edinburgh); YS Lim (Dr Gray’s Hospital; Elgin)

*United States*: S Agarwal (Duke University Hospital; Durham); P Petrone (NYU Langone Hospital–Long Island; New York); D Stephens (Mayo Clinic; Rochester); N Starr (Zuckerberg San Francisco General Hospital; San Francisco); A Teichman (Rutgers RWJ Hospital; New Brunswick); C Dodgion (Froedtert & the Medical College of Wisconsin; Milwaukee); C Wolff (Cleveland Clinic Akron General; Akron); T Egodage (Cooper University Hospital; Camden); J Brady (Chippenham Hospital; Richmond); J Brown (University of Pittsburgh Medical Center; Pittsburgh); D Leon (University of California Davis Medical Center; Sacramento)

*Uruguay*: A Pienovi (Hospital de Clínicas Dr. Manuel Quintela; Montevideo)

*Yemen*: R Saleh (Al Thawrah Hospital Ibb; Ibb)

***Collaborators***

*Albania*: K Doçi, E Bregaj (University Trauma Hospital; Tirana)

*Algeria*: J Mansouri, B Tabeti (EHU-1st November 1954; Oran)

*Argentina*: M Titarelli, MM Avalos Barraza , M Sánchez (Hospital de Urgencias de Córdoba; Córdoba); E Caldani, A Giavarini (Hospital de Emergencias Dr Clemente Alvarez; Rosario)

*Australia*: C Groombridge, E Ban (The Alfred Hospital; Melbourne)

*Bahrain*: A Abdulla (Salmaniya Medical Complex Government Hospital; Manama)

*Belarus*: V Bereshchenko, P Tereshchenko (Gomel State Medical University; Gomel)

*Brazil*: I Marcos, R Lima (Hospital Regional Dom Moura; Garanhuns)

*Cameroon*: N Nwenasi (Yaoundé Central Hospital; Yaoundé); E Aloys (Centre des Urgence de Yaoundé; Yaoundé)

*Canada*: H Uchino, JR Grushka, W Davalan (McGill University Health Centre; Montreal)

*China*: C Chen, F Ge (Shanghai General Hospital, Shanghai Jiao Tong University School of Medicine, Shanghai); K Lu (The Affiliated Changzhou No.2 People's Hospital of Nanjing Medical University; Changzhou); J Zhang (The People's Hospital of Yuyao City, The Affiliated Yangming Hospital of Ningbo University; Ningbo); X Liu, X Li (Beijing Jishuitan Hospital, Capital Medical University; Beijing); Z Qi (Hebei Medical University Third Hospital; Shijiazhuang); N Wang, J Tang, S Wang, F Gao, Y Lu, H Du (The Second Affiliated Hospital of Zhengzhou University; Zhengzhou); C Wu, H Fu (Zhongda Hospital, Southeast University; Nanjing); J Liu (Tianjin Medical University General Hospital; Tianjin); T Chen (Sir Run Run Shaw Hospital, Zhejiang University School of Medicine; Hangzhou); M Du, J Guan (Dongguan Eastern Central Hospital, The Sixth Affiliated Hospital of Jinan University; Dongguan); Q Lu, Y Li, J Li, Q Wang, W Luo (The Second Affiliated Hospital of Kunming Medical University; Kunming); K Wang (Affiliated Jinhua Hospital, Zhejiang University School of Medicine; Jinhua); H Zhang, J Dong (Shanghai Pudong New Area Gongli Hospital; Shanghai)

*Colombia*: S Gelvez, K Reyes (Hospital Universitario del Valle; Cali)

*Democratic Republic of the Congo*: D Tsongo, J Muhoza (Hôpital de Kyeshero; Goma); A Mirindi, JR Birindwa (Hôpital Provincial Général de Référence de Bukavu; Bukavu)

*Egypt*: H Aboelfadl, A Elasad, S Elsheikh, F Elsaied, M Atef, R Elnour, N Elbaloula, EA Shanab, M Adres, O Mohamed, S Adam (Al-Ahrar Zagazig Teaching Hospital; Zagazig); O Younes, T Elboraay, O Abdelfattah, MA Elfadali, A Jader, A Ziada, K Sarhan, M Sherif , R Gomaa, R Mohamed, E Fouda, A Elshaboury, M Alshraiedeh, H Elhadidi, A Eldiasti, M Ahmed, E Elkoury, H Abdelhady, M Amasha, A Elbadrawy, M Nassif, M Hamed (Mansoura Specialized Hospital; Mansoura); D Zahran, S Abdelaal, OS Abdelfattah, W Shehada, M Alsharif, M Elnadi, O Sharaf, M Elgliand, M Badr, A Hegazi, A Gohar, A Elshal, M Abdelhady, M Saadawi, M Mohammed, EY Salem (Delta Hospital; Mansoura); M Madany (Aswan University Hospital; Aswan); H Mansour, A Ashour, A AbuSuliman, K Tolba, MS Elgendy, M Ezz, M Marei, HI Taha, I Younes, A Abouammar (Tanta University Hospital; Tanta); R Wael, Asmaa Elmorshdy, Aesha Elmorshdy, AM Ibrahim, A El-Borollosy, S Elmorshdy, A Mohamed, A Adel, NH El-Saeed, M Essam, EAK Abdelraheem, M Shaapan, M Salah, E Saber, MA Ibrahim, R Mamdouh, AMM Mohamadin, F Moharb (Mataria Teaching Hospital; Cairo); O Shaqran, Z Selim, Y Tanas, M Khalil, B Eldin, Y Gaber, A Ibrahim, D Bekhit, B Mohamed, A Farrag (Alexandria Main University Hospital; Alexandria); M Saadawi, H Mahfouz (Beni Suef Health Insurance Hospital; Beni Suef); N Sayed, M El Mahrouki, A Amgad, D Elmagdoub, S Paulo (Kasr Al Ainy Hospital; Cairo)

*Ethiopia*: B Gebremedhin, A Eticha (Arsi University Asella Referral and Teaching Hospital; Asella); B Bayissa, K Urgessa, B Tasew (Adama Hospital Medical college; Adama City); Y Yilma, LG Mude, O Tilahun (Jimma University Medical Centre; Jimma); L Buta, AG Mideksa, T Gemechu (ALERT Comprehensive Specialized Hospital; Addis Ababa); A Tilahun, Y Degefu (St. Paul’s Hospital Millenium Medical College; Addis Ababa)

*Ghana*: F Quenin, E Acquah, I Abdull-Karim, C Takyi (Holy Family Catholic Hospital; Techiman); G Aryee, T Wordui, A Bowan, P Kumassah, N Adu-Aryee, N Naalane, F Dedey, J Nsaful (Korle Bu Teaching Hospital; Accra); O Ekor, G Rahman, M Nortey, R Baidoo, M Amoako-Boateng, D Enti, K Agyen-Mensah, E Quartson, T Agyen, E Ofori, P Mensah, V Kudoh, D Arthur, P Maison (Cape Coast Teaching Hospital; Cape Coast); F Akum, F Owusu (Sunyani Teaching Hospital; Sunyani); N Affram, D Tamatey (Ho Teaching Hospital; Ho)

*Greece*: C Sarakatsianou, D Papaspyrou (General University Hospital of Larissa; Larissa); A Antzoulas, K Kitsou, V Garantzioti, (University Hospital of Patras; Patras); V Leivaditis (General Hospital of Aigio; Aigio); A Vouchara, K Katsiafliaka (AHEPA University Hospital of Thessaloniki; Thessaloniki)

*Guatemala*: S Morales, E Galindo, A Meza, M Colón, E Cardona (Hospital General San Juan de Dios; Guatemala City)

*Haiti*: K Louis, R Osias, C Lominy, A Capois (Hôpital Universitaire La Paix; Port-au-Prince)

*India*: SA Khan, V Verma (Maulana Azad Medical College; New Delhi); S Amin, A Gaikwad (Seth GS Medical College and KEM Hospital; Mumbai)

*Italy*: V Tonini, M Cervellera (Presidio Ospedaliero Centrale - SS. Annunziata; Taranto); M Fumagalli, M Zizzo, D Luppi, H Yu, L Di Donato (Azienda USL - IRCCS di Reggio Emilia; Reggio Emilia); F Leo, C Cecchi, G Ripamonti, B Pesi, L Piombetti, M Pagani, G Pascale, S Di Salvatore, C Tasca, S Giannessi, R De Vincenti, E Monati (San Jacopo Hospital, Pistoia); F Renzi (Ospedale S. Maria alla Gruccia; Montevarchi); L Vacca, F Matarazzo, D Perini, A Di Bella, L Fortuna (Azienda Ospedaliero Universitaria Careggi; Firenze); M Rottoli, M Binetti (IRCCS Azienda Ospedaliero - Universitaria di Bologna; Bologna); M Tescione, G Sera, N Pellicano, S Pangallo (Grande Ospedale Metropolitano; Reggio Calabria); E Ballauri, M Santarelli (AOU Cittá della Salute e della Scienza; Torino); S Cimbanassi, S Cioffi, G Curreri (ASST Grande Ospedale Metropolitano Niguarda; Milan); M Ceolin, D Del Fabbrio, S Giudici, M Cecconi (IRCCS Istituto Clinico Humanitas; Milan); T Sinicropi, C Mazzeo (AOU Policlinico G Martino; Messina)

*Japan*: K Sato (Ohta Nishinouchi Hospital; Fukushima)

*Kenya*: K Otoki, D Baraka (Tenwek Hospital; Tenwek)

*Malaysia*: RMZ Ang, MNF Zulkifli, KC Sheng, MTY Wong, N Aziz, PJH Lim, CCE Koay (Universiti Malaya Medical Centre; Kuala Lumpur); YX Teoh, I Chik (Hospital Canselor Tuanku Muhriz UKM; Kuala Lumpur); Z Zakaria, MHS Satar, MR Mazlan (USM Specialist Hospital, Universiti Sains Malaysia; Kubang Kerian)

*Mexico*: L Bravo-Cuéllar, J Orozco-Camacho, A Nava-Franco, M Ibarra-Tapia, F López-Ortega, F Romo-Pérez, R Contreras-Arias, M Alejo-Rivera (Hospital Civil de Guadalajara Fray Antonio Alcalde; Guadalajara); SJ Vázquez-Sánchez, C Fuentes-Orozco (Centro Médico Nacional de Occidente IMSS; Guadalajara)

*New Zealand*: A McCombie, J Dasril, Y Teo (Christchurch Hospital; Christchurch)

*Niger*: AA Fagbenro, K Shafer (SIM Galmi Hospital; Galmi)

*Nigeria*: L Iji, S Gana, M Bashir, A Ajayi, I Gundu, GD Mukoro, L Ukwubile, C Okeke, A Jimoh, V Nduka (Ahmadu Bello University Teaching Hospital; Zaria); K Bwala, A Ningi (Abubakar Tafawa Balewa University Teaching Hospital; Bauchi); S Oriakhi, H Odion-Obomhense (Delta State University Teaching Hospital; Oghara); S Ekpemo, K Okpokiri (Federal Medical Centre; Umuahia); AA Makama, H Aminu, Z Ahmad, M Mustapha, O Segunfunmi (Federal Medical Center Nguru; Nguru); E Boladuro, U Eni, C Obi, NL Kwentoh (Alex Ekwueme Federal University Teaching Hospital; Abakaliki); D Idowu, M Magbagbeola (Ekiti State University Teaching Hospital; Ado Ekiti); O Adolphus, E Oriabure (The Hills Medical Center; Benin City); S O Fatudimu; O Oloruntoba (Federal Teaching Hospital Ido-Ekiti; Ido Ekiti); R Eghonghon, S Omorogbe (University of Benin Teaching Hospital; Benin City)

*Pakistan*: S Shaikh, AK Narsani, I Ujjan, A Munir, AI Memon, F Hameed, S Khatoon, A Talpur, S Kumar, A Yousfani, N Dal, S Naz, M Akbar, AM Bhatti, N Amir, SA Khaskheli (Liaquat University of Medical and Health Sciences; Jamshoro); N Iqbal, A Aamir, G Shamsi, G Awais, I Tasleem, N Iodhi, I Ahmed, R Fatima, S Asif, H Haroon, A Jawaid, J Muneer, H Ahmed, M Washdil, A Hilal, M Ishaq, S Ialani, Y Kumar, MN Shehzad, S Nadeem, N Ahmed, S Ahmed, S Gulzar (Indus Hospital and Health Network; Karachi); G Khan, Z Jaffer, A Gul, M Khan, A Faraz, M Obaid (Lady Reading Hospital; Peshawar); H Pirhay, K Faheem, M Shafique, R Nafees (PAF Hospital Base Faisal; Karachi)

*Paraguay*: LC Soares Barboza de Toledo, AL Silva de Sousa (Hospital de Trauma Prof. Dr. Manuel Giagni; Asunción)

*Occupied Palestinian Territories*: B Qneiby, R Jabari, R Farash (Al-Ahli Hospital; West Bank); M Oweidat (Hebron Governmental Hospital; West Bank); R Matar, M Shaldan (Nasser Hospital; Gaza)

*Peru*: C Picasso-Arias (Clinica Internacional, Lima)

*Portugal*: C Strong, F Feliciano, L dos Santos, D Monteiro (Unidade Local de Saúde de Lisboa Ocidental; Lisboa); S Alves, D da Cruz (Unidade Local de Saúde São João; Porto)

*Romania*: B Oprita, E Dumitru (Clinical Emergency Hospital of Bucharest; Bucharest)

*Russia*: L Lichman, O Davydova, P. Andreev (Clinics of Samar Medical University; Samara)

*Saudi Arabia*: R Alyahya, AM Alrwais, NH Almadi, N AlShahwan, M Aladawi, SH Aldeligan, A Alotaibi (King Saud University Medical City; Riyadh)

*Singapore*: J Lee, S Gunasekaran, MW Ong, DJK Lee, WW Lim (Khoo Teck Puat Hospital; Singapore); LT Teo, RL Tan (Tan Tock Seng Hospital; Singapore)

*Somalia*: AS Hashi, AA Omar, AN Mohamed, AM Abdi (Mogadishu Somali-Türkiye Recep Tayyip Erdoğan Training and Research Hospital, Mogadishu); AH Salad (Kakaal Hospital; Mogadishu); AE Abdishakur (Somali-Sudanese Specialized Hospital; Mogadishu)

*South Africa*: F Ganchi, S Naidoo, K Moodley, H Wain (Inkosi Albert Luthuli Central Hospital; Durban); N Reddy (Ngwelezana Hospital; Empangeni); N Laher, D Wineberg, R Pretorius, R Pswarayi, E Laney, O Lusawana, A Mushtaq, I Bogiages, F Viljoen (Chris Hani Baragwanath Academic Hospital; Johannesburg); F Mohammed, G Jacks, L Mohlala, C Nyatsambo, S Mathibela, A Nortje, S Makhadi, T Pratt, K de Kock (Charlotte Maxeke Johannesburg Academic Hospital; Johannesburg); MQ Patel, M Parker, JJP Buitendag, GV Oosthuizen (Tygerberg Hospital; Cape Town)

*Spain*: C Martínez de Carneros, S Quinto Llopis (Hospital General Universitario de Elche; Alicante); L Cruzado, A Sainz-Lete, B Estraviz-Mateos, JC Zevallos-Quiroz (Urduliz Hospital; Bizkaia); I Augusto Ponce, A Garcia Domínguez, A Lizarazu Perez, A Rodriguez Gonzalez (Hospital Universitario Donostia; San Sebastián); A Muñoz-Campaña, A Campos-Serra (Parc Taulí Hospital Universitari; Sabadell)

*Sri Lanka*: L Bandara, K Gunasekara, G Jayarathne, Y Arachchi, M Priyangani, S Wimalge (District General Hospital Kegalle; Kegalle); RSC Desman, K Gunarathne (District General Hospital; Dambulla); G Wimalasena, V Rohana, S Ranathunga (National Hospital of Sri Lanka; Colombo); S Harikrishanth, J Jeyaruban (Teaching Hospital Batticaloa; Batticaloa)

*Sudan*: M Mohammed, A Mohamed, L Zeinalabedeen, A Mahmoud (Atbara Teaching Hospital; Atbara); M Mohamed (Prince Digna Referral Hospital; Port Sudan); M Eltahir, G Ahmed, M Ahmed (Al Hasahissa Teaching Hospital; Al-Hasahisa); IAO Mohammed, SAA Ibrahim, EAH Aziz (Bashair Teaching Hospital; Khartoum); M Homida, FS Mahdi (Port Sudan Teaching Hospital; Port Sudan); M Issak, A Mohammed (Dongola Specialized Hospital; Dongola); M Hafiz, H Makki (Kassala Teaching Hospital; Kassala City); N Awad, A Elhassan (Alnao Teaching Hospital; Omdurman City); M Amin, A Daffalla, A Omer (Sinnar Teaching Hospital; Sinnar City); M Alhadi, M Mostafa, O Eljizoly (Gadarif Teaching Hospital; Gadarif); A Musa, M Abdallah (Ad-Damazin Teaching Hospital; Ad-Damazin); G Fakhri, A Ahmed, A Mohammed (New Halfa Teaching Hospital; New Halfa)

*Sweden*: M Kollind, S Marchesi (Skåne Universitetssjukhus Malmö; Malmö)

*Syria*: A Aldirani, A Almahjaa, M Abdulkareem, E Kallas, A Alfandi, A Hejazi, B Alnaser, A Alnaser, J Sandouk (Damascus Hospital; Damascus); S Sara, K Ballan, H Joha (Al Mouwasat Hospital; Damascus); S Kassis, W Abboud, M Ahmad, A Hamdan (Homs University Hospital; Homs)

*Thailand*: K Chandacham, T Jirapongcharoenlap, N Chotirosniramit (Maharaj Nakorn Chiang Mai Hospital; Chiang Mai)

*Tunisia*: R Trigui, O Gaidi (Menzel Bourguiba Hospital; Bizerte); A Saidani, A Belhaj (Mahmoud Matri Hospital; Ariana); H Zebda, A Menif, R Khelili (Beja Hospital; Beja)

*Türkiye*: Ç Bayır, Ö Acar, E Bozlakoğlu (Marmara University School of Medicine; Istanbul); E Yavuz, G Alici, S Meric, N Bugdayci, A Sayar, A Ergin, A Saylar, A Barcin, Y Altinel, O Gulcicek, O Cakir (Bagcilar Training and Research Hospital; Istanbul); H Ozsahin, C Ersavas (Uskudar University Faculty of Medicine, Memorial Hospital; Istanbul); GK Aydoğdu (Bandirma Training and Research Hospital; Balıkesir); K Saraçoğlu, N Dundar (Kocaeli City Hospital; Izmit); K Eyuboglu, R Tekcan, M Bodur, M Aktas, B Erdem, A Calik, A Kodalak, A Oruc, M Usta, B Alkas, M Rahimi, A Cekic, D Pehlivan, I Rizaoglu, A Mwinyi, B Canakci, M Shehada, S Topaloglu (Karadeniz Technical University Farabi Hospital; Ortahisar); A Karaaslan, G Ercan, Y Poyrazoğlu, M Çuhadar, Ö Özkan (Sultan Abdülhamid II Han Training and Research Hospital; Istanbul); R Ağcabay, G Tuncer, S Farsak, C Tuğmen, N Polat, E Kebapçı, M Yıldırım, N Göret, M Gündal, S Ünlü, E Tekel (Bakircay University Cigli Education and Research Hospital; Izmir); A Özpek, H Tosun (University of Health Sciences Umraniye Training and Research Hospital; Istanbul); B Yeşilova, K Dikmen, H Göbüt, A Yavuz (Gazi University Hospital; Ankara); S Zeren, Y Sönmez (Kütahya City Hospital; Kütahya); T Gulsen, M Zenciroglu (Sultanbeyli State Hospital; Istanbul)

*Ukraine*: P Kyrylo, P Kostiantyn, P Ivan (Kharkiv Regional Clinical Hospital; Kharkiv)

*United Kingdom*: P Orchard, J Fyfe (Southmead Hospital; Bristol); H Dowell, O Braun, M Creed, P Strong, F Sweeney, N Mitchell, I McClure, D Parry, O Gbadegesin, E Carrington-Windo, M McKenna, S Mundell, L Hall, S Gasson, E Crudge, A Eglinton (University Hospital of Wales, Cardiff); R Davenport, P Vulliamy, ZB Perkins, O Ugas (Royal London Hospital; London); L Holt, H Jenkinson, J Tan (University Hospital Aintree ; Liverpool); G Ramsay, O Adepoju, R Cummine, S Tariq, A Mohammad, L Wilson (Aberdeen Royal Infirmary; Aberdeen); A Musbahi, R Coates (Sunderland Royal Hospital; Sunderland); SJ Horne, N Preda, F Luvisetto (University Hospital Plymouth; Plymouth); Z Zhang, I Saqib, G Matzakanis, P Pearce (John Radcliffe Hospital; Oxford); N Sánchez-Thompson, C Scurr, A Bernstein (St Mary’s Hospital; London); MS Gonsalves, MS Hoque-Uddin, I Abbott, O Dada (Queen Elizabeth Hospital; Birmingham); S Jamil, H Read, D Horner, R Doonan, A Stafford (Salford Royal Hospital; Salford); C Battle (Morriston Hospital; Swansea); R Thavayogan, O Quinn (Norfolk and Norwich University Hospital; Norwich); M Powar, S Ling, S Gourgiotis (Addenbrooke’s Hospital; Cambridge); H Shinwari (St George’s University Hospital; London); S Mohandas (North Middlesex University Hospital; London); M Eldoadoa, R Ismail (Manchester Royal Infirmary; Manchester); G Melia, N Gandhi, L Blackburn (Nottingham University Hospitals; Nottingham); T Merchant (Royal Victoria Hospital; Belfast); J Robinson, S Mackie (Royal Infirmary of Edinburgh; Edinburgh); YS Wong, Q Lee (Dr Gray’s Hospital; Elgin)

*United States*: D Moris, CP Nicholson Jr., S Provencher, J Cook (Duke University Hospital; Durham); G Baltazar, K Cordero-Bermudez (NYU Langone Hospital - Long Island; New York); LE Walker, MK Abou Chaar (Mayo Clinic; Rochester); R Koch, K Faktor, A Chang (Zuckerberg San Francisco General Hospital; San Francisco); Z Englert, C Kyaw, N Pirozzi, L Moko, B Chernock, E Marshall (Rutgers RWJ Hospital; New Brunswick); JA Gellings (Froedtert & the Medical College of Wisconsin; Milwaukee); J Krizo, J Molinari (Cleveland Clinic Akron General; Akron); E Hancin, I Armento (Cooper University Hospital; Camden); P Hu, R Uhlich, E Barnes (Chippenham Hospital; Richmond); A Rawal, O Falade (University of Pittsburgh Medical Center; Pittsburgh); D Nishijima, D Leshikar (University of California Davis Medical Center; Sacramento)

*Uruguay*: E Delgado (Hospital de Clínicas Dr. Manuel Quintela; Montevideo)

*Yemen*: S Al Wageeh, A Al Yafrosi (Al Thawrah Hospital Ibb; Ibb)

***Data Validators***

*Haiti*: L Minthor (Hopital Universitaire La Paix, Port-au-Prince)

*Italy*: S Cardelli (IRCCS Azienda Ospedaliero - Universitaria di Bologna, Bologna)

*Malaysia*: W Chiew Meng (Universiti Malaya Medical Centre, Kuala Lumpur); S Johan (USM Specialist Hospital, Universiti Sains Malaysia; Kubang Kerian)

*Nigeria*: IE Ihedoro (Federal Medical Centre, Abia)

*Pakistan*: A M Waryah (Liaquat University of Medical and Health Sciences, Jamshoro)

*Peru*: D Chavez (Clinica Internacional, Lima)

*Sri Lanka*: T Manivannan (Teaching Hospital Batticaloa, Batticaloa)

*Sudan*: M A M Abdalla (Dongola Specialized Hospital, Dongola)

*Tunisia*: A Itaimi (Menzel Bourguiba Hospital, Bizerta)

*Türkiye*: TK Uprak (Marmara University School of Medicine, Istanbul); M Ulusahin (Karadeniz Technical University Farabi Hospital, Trabzon)

*United Kingdom*: CV Riley (Salford Royal Hospital, Salford); H Hussein (North Middlesex University Hospital, London); T Edmiston (Addenbrookes Hospital, Cambridge); L Nicol (Dr Gray's Hospital, Elgin)

**Supplementary Material 2 - Directed Acyclic Graph**


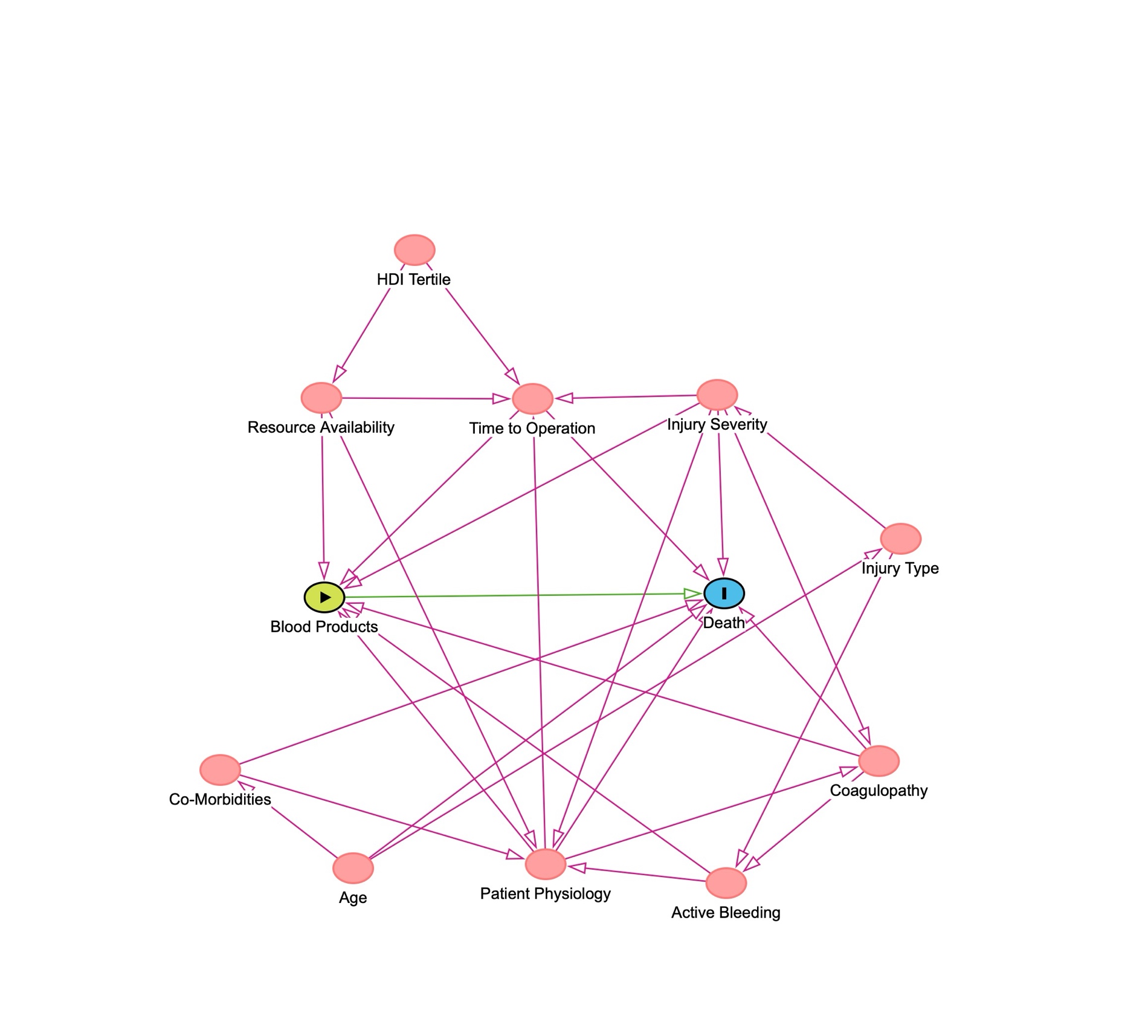


**Supplementary Material 3 –** **Patient factors and blood product availability across the entire cohort**

|  | **Lower HDI**  (n=563) | **Middle HDI**  (n=713) | **Upper HDI**  (n=492) | **Total**  (n=1768) | **p-value** |
| --- | --- | --- | --- | --- | --- |
| Age (years), mean (SD) | 28.6 (12.7) | 34.5 (15.2) | 40.1 (17.6) | 34.2 (15.8) | <0.0001 |
| Male sex, n (%) | 488 (86.7) | 630 (88.3) | 393 (79.9) | 1511 (85.5) | 0.0013 |
| Mechanism of injury  - Blunt, n (%)  - Penetrating, n (%) | 226 (40.1)  337 (59.9) | 294 (41.2)  419 (58.8) | 294 (59.8)  198 (40.2) | 814 (46.0)  954 (54.0) | <0.0001 |
| Systolic Blood Pressure on arrival (mmHg), mean (SD) | 105 (24) | 113 (27) | 110 (30) | 109 (28) | <0.0001 |
| ISS, median (IQR) | 9 (4-16) | 9.5 (8-22) | 16 (9-27) | 9 (5-20) | <0.0001 |
| Time to operate (hours), mean (SD) | 17.1 (62.1) | 14.7 (17.3) | 12.7 (52.1) | 15.0 (45.8) | <0.0001 |
| AIS ≥4 (severe injury), n (%) | 133 (23.6) | 221 (31.0) | 193 (39.2) | 547 (30.9) | <0.0001 |
| Physiological signs of shock, n (%) | 144 (25.6) | 228 (32.0) | 192 (39.0) | 674 (38.1) | <0.0001 |
| Intraoperative blood loss >1000ml, n (%) | 170 (31.3) (n=543) | 275 (39.5)  (n=696) | 193 (42.7)  (n=452) | 638 (37.7)  (n=1691) | 0.0004 |
| Centre blood product availability  - All of the time  - Most of the time  - Some of the time | 315 (56.0)  228 (40.5)  20 (3.6) | 397 (55.7)  192 (26.9)  124 (17.4) | 463 (94.1)  29 (5.9)  0 (0) | 1175 (66.5)  449 (25.4)  144 (8.1) | <0.0001 |

**Supplementary Material 4 - Histogram of patient FFP:PRBC ratio across the entire cohort**

**
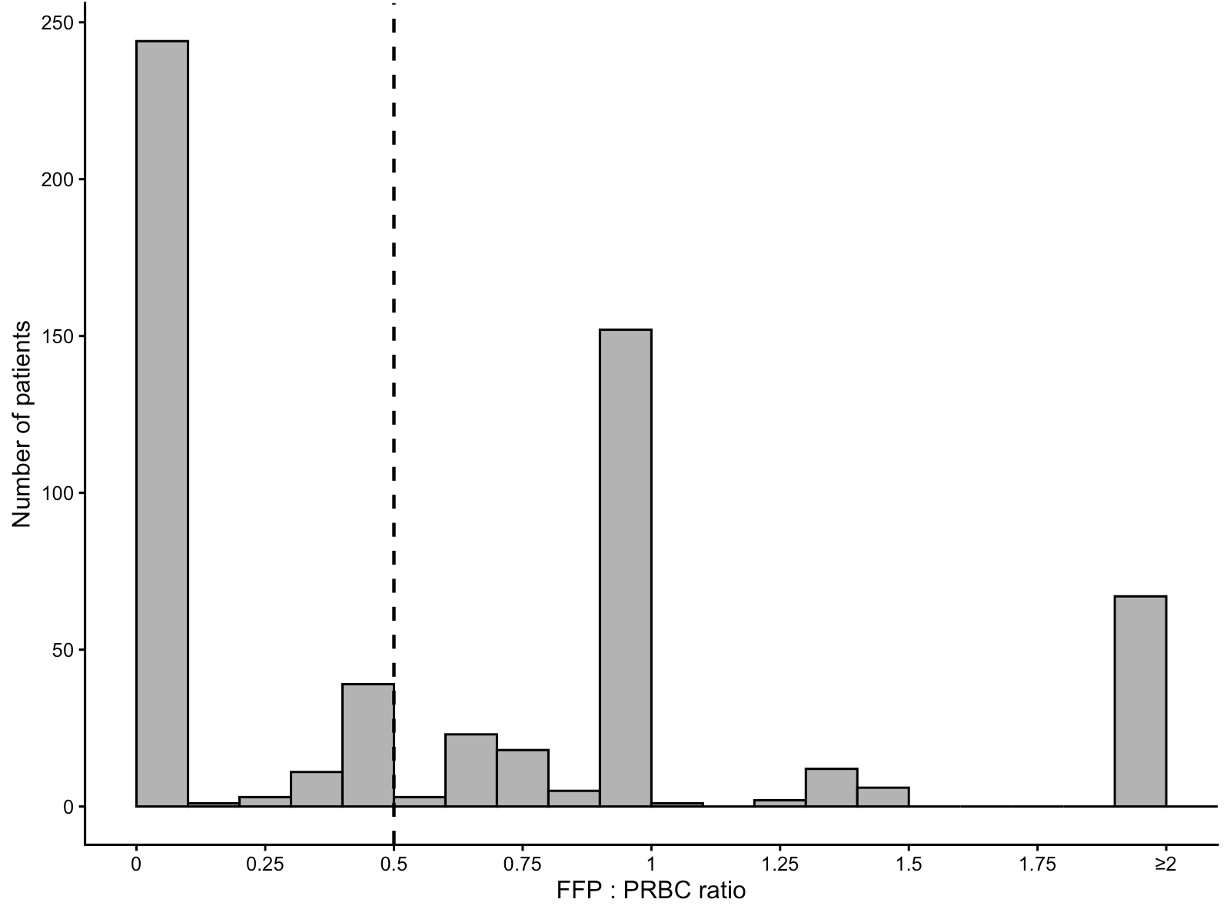
**

**Supplementary Material 5 – Forest plot of multivariate analysis for 30-day mortality, comparing high ratio and low ratio cohorts, with the addition of blood product transfusion volume as a co-variate**

**
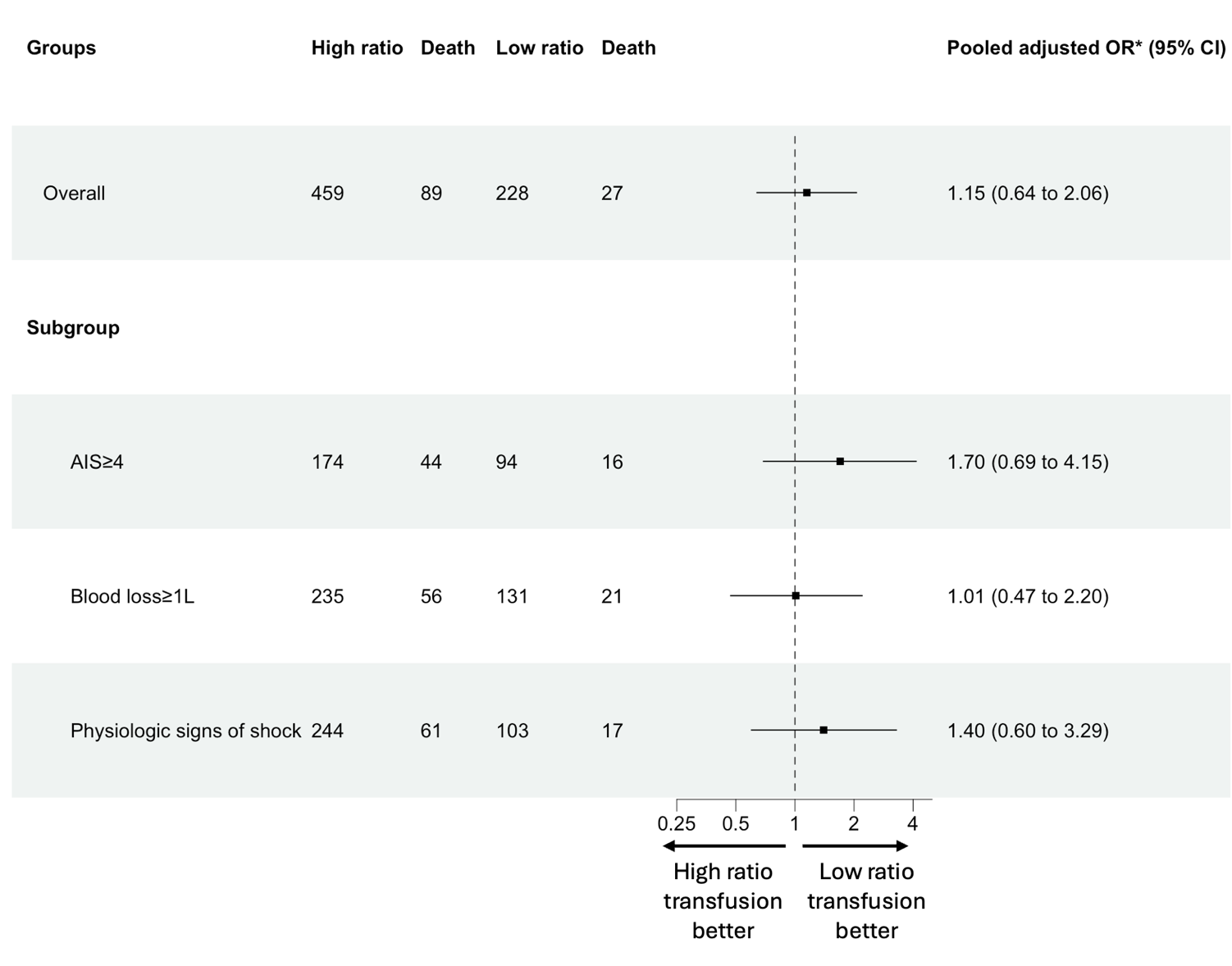
**

**Supplementary Material 6 -** **STROBE Guidelines Checklist**

|  | **Item No** | **Recommendation** | | **Page No** |  |
| --- | --- | --- | --- | --- | --- |
| Title and abstract | 1 | (a) Indicate the study’s design with a commonly used term in the title or the abstract | | 1 |  |
|  |  | (b) Provide in the abstract an informative and balanced summary of what was done and what was found | | 3 |  |
| Introduction | | | | | |
| Background/rationale | 2 | Explain the scientific background and rationale for the investigation being reported | | 5 |  |
| Objectives | 3 | State specific objectives, including any prespecified hypotheses | | 5 |  |
| Methods | | | | | |
| Study design | 4 | Present key elements of study design early in the paper | | 6 |  |
| Setting | 5 | Describe the setting, locations, and relevant dates, including periods of recruitment, exposure, follow-up, and data collection | | 6 |  |
| Participants | 6 | (a) Give the eligibility criteria, and the sources and methods of selection of participants. Describe methods of follow-up | | 6 |  |
|  |  | (b) For matched studies, give matching criteria and number of exposed and unexposed | | N/A |  |
| Variables | 7 | Clearly define all outcomes, exposures, predictors, potential confounders, and effect modifiers. Give diagnostic criteria, if applicable | | 6 |  |
| Data sources/ measurement | 8 | For each variable of interest, give sources of data and details of methods of assessment (measurement). Describe comparability of assessment methods if there is more than one group | | 6 |  |
| Bias | 9 | Describe any efforts to address potential sources of bias | | 6-7 |  |
| Study size | 10 | Explain how the study size was arrived at | | 6 |  |
| Quantitative variables | 11 | Explain how quantitative variables were handled in the analyses. If applicable, describe which groupings were chosen and why | | 6-7 |  |
| Statistical methods | 12 | (a) Describe all statistical methods, including those used to control for confounding | | 6-7 |  |
|  |  | (b) Describe any methods used to examine subgroups and interactions | | 7 |  |
|  |  | (c) Explain how missing data were addressed | | 7 |  |
|  |  | (d) If applicable, explain how loss to follow-up was addressed | | N/A |  |
|  |  | (e) Describe any sensitivity analyses | | N/A |  |
| Results | | |  | | |
| Participants | 13 | (a) Report numbers of individuals at each stage of study—eg numbers potentially eligible, examined for eligibility, confirmed eligible, included in the study, completing follow-up, and analysed | | 8-9 |  |
|  |  | (b) Give reasons for non-participation at each stage | | 8-9 |  |
|  |  | (c) Consider use of a flow diagram | | Fig 3 |  |
| Descriptive data | 14 | (a) Give characteristics of study participants (eg demographic, clinical, social) and information on exposures and potential confounders | | 8-9 |  |
|  |  | (b) Indicate number of participants with missing data for each variable of interest | | 8-9 |  |
|  |  | (c) Summarise follow-up time (eg, average and total amount) | |  |  |
| Outcome data | 15 | Report numbers of outcome events or summary measures over time | | 8-9 |  |
| Main results | 16 | (a) Give unadjusted estimates and, if applicable, confounder-adjusted estimates and their precision (eg, 95% confidence interval). Make clear which confounders were adjusted for and why they were included | 8-9 | | |
|  |  | (b) Report category boundaries when continuous variables were categorized | 8-9 | | |
|  |  | (c) If relevant, consider translating estimates of relative risk into absolute risk for a meaningful time period | N/A | | |
| Other analyses | 17 | Report other analyses done—eg analyses of subgroups and interactions, and sensitivity analyses | 9 | | |
| Discussion | | |  |  |  |
| Key results | 18 | Summarise key results with reference to study objectives | 10 | | |
| Limitations | 19 | Discuss limitations of the study, taking into account sources of potential bias or imprecision. Discuss both direction and magnitude of any potential bias | 111 | | |
| Interpretation | 20 | Give a cautious overall interpretation of results considering objectives, limitations, multiplicity of analyses, results from similar studies, and other relevant evidence | 10-11 | | |
| Generalisability | 21 | Discuss the generalisability (external validity) of the study results | 11 | | |
| Other information | | |  |  |  |
| Funding | 22 | Give the source of funding and the role of the funders for the present study and, if applicable, for the original study on which the present article is based | 2 | | |
